# Weighted Gene Co-Expression Network Analysis Reveals Dysregulation of Mitochondrial Oxidative Phosphorylation in Eating Disorders

**DOI:** 10.3390/genes9070325

**Published:** 2018-06-28

**Authors:** Liulin Yang, Yun Li, Turki Turki, Huizi Tan, Zhi Wei, Xiao Chang

**Affiliations:** 1 College of Electrical Engineering, Guangxi University, Nanning 530004, Guangxi, China; yang03667@126.com; 2 Department of Biostatistics and Epidemiology, The Perelman School of Medicine, University of Pennsylvania, Philadelphia, PA 19104, USA; yli0131@gmail.com; 3 Department of Computer Science, King Abdulaziz University, P.O. Box 80221, Jeddah 21589, Saudi Arabia; tturki@kau.edu.sa; 4 School of Food Science and Technology, Jiangnan University, Wuxi 214122, Jiangsu, China; huizi.tan@yahoo.com; 5 Department of Computer Science, New Jersey Institute of Technology, Newark, NJ 07102, USA; 6 The Center for Applied Genomics, Children’s Hospital of Philadelphia, Philadelphia, PA 19104, USA

**Keywords:** eating disorders, weighted gene co-expression network analysis

## Abstract

The underlying mechanisms of eating disorders (EDs) are very complicated and still poorly understood. The pathogenesis of EDs may involve the interplay of multiple genes. To investigate the dysregulated gene pathways in EDs we analyzed gene expression profiling in dorsolateral prefrontal cortex (DLPFC) tissues from 15 EDs cases, including 3 with anorexia nervosa (AN), 7 with bulimia nervosa (BN), 2 AN-BN cases, 3 cases of EDs not otherwise specified, and 102 controls. We further used a weighted gene co-expression network analysis to construct a gene co-expression network and to detect functional modules of highly correlated genes. The functional enrichment analysis of genes in co-expression modules indicated that an altered mitochondrial oxidative phosphorylation process may be involved in the pathogenesis of EDs.

## 1. Introduction

Eating disorders (EDs), including anorexia nervosa (AN), bulimia nervosa (BN), and binge-eating disorder are psychiatric diseases that cause severe disturbances to a person’s eating behavior. Patients with EDs may also have other illnesses, such as anxiety, depression, and substance abuse. Although twin studies have suggested that EDs have a strong genetic background with a few candidate genes of AN and BN reported previously [1,2,3,4,5,6,7], two recent genome-wide association studies (GWAS) of more than 5500 AN cases and 21,000 controls still failed to identify a genome-wide significant signal [8,9]. This suggests that there is a complex genetic mechanism underlying EDs which may involve multiple functional linked genes from the same biological pathway.

The weighted gene co-expression network analysis (WGCNA) has been successfully applied to multiple studies of the transcriptome changes in psychiatry disorders, such as autism, bipolar disorder, and schizophrenia [10,11,12]. For example, dysregulation of the innate immune response or inflammatory-related gene pathways has been reported in multiple studies of autism and schizophrenia [12,13] However, to our best knowledge, no transcriptome study has been performed on patients with EDs using WGCNA.

In this paper, we studied gene expression profiling in the dorsolateral prefrontal cortex (DLPFC) tissues of diseased individuals with an ED and identified candidate genes and pathways that are involved in the pathophysiology of EDs. We further built a gene co-expression network and detected functional modules of highly correlated genes. Our study implicates the mitochondrial oxidative phosphorylation process in the pathogenesis of EDs.

## 2. Materials and Methods

### 2.1. Gene Expression Data

Gene expression data of the DLPFC from postmortem tissue on 15 diseased patients with EDs and 102 diseased non-psychiatric controls were obtained from the GEO (Gene Expression Omnibus) repository (accession number: GSE60190) [14]. All patients with EDs met the DSM-IV criteria for one or more axis-I diagnoses of an ED in their lifetime (AN, BN, or ED not otherwise specified). Clinical data included family informant interviews with next-of-kin, retrospective psychiatric record reviews, and data from a medical examination, including cause/manner of death. All of these factors were summarized in a psychiatric narrative format and reviewed by two board-certified psychiatrists. The non-psychiatric controls were free from psychiatric diagnoses and substance abuse, according to the DSM-IV. Every individual in the control cohort was subject to toxicology screening to exclude acute drug and alcohol intoxication/use at the time of death, and all fetal tissues were screened for possible in utero drug exposure. Details of the subjects used in this study are summarized in Table S1. The gene expression levels of all subjects were generated on Illumina HumanHT-12 v3 microarrays (one-color, Illumina, San Diego, CA, USA) and normalized with background correction and variance-stabilizing transformation. This was followed by quantile normalization [15]. Differentially expressed genes were calculated by the LIMMA package in R language [16].

### 2.2. Weighted Gene Co-Expression Network Analysis

The weighted gene co-expression network analysis is widely used for constructing a co-expression network based on scale-free topology and detecting co-expression modules of genes with similar patterns of expression. At first, a matrix of correlations between all gene pairs was calculated using the expression values and then converted into an adjacency matrix using the power function, *β*, where the connection strength between two genes, *x_i_* and *x_j_*, was defined as *a_ij_* = |*cor*(*x_i_*, *x_j_*)|·*β*. In this study, *β* was set as 12 to allow the resulting adjacency matrix to approximately fit a scale-free topological feature. According to a previously proposed model-fitting index [17], the model-fitting index of a perfect scale-free network is 1. Here, 12 was the minimum value of *β* (see Figure S1) required to make the model-fitting index above 0.8 [17].

The adjacency matrix was next transformed into a topological overlap matrix (TOM), which captured not only the direct interaction between two genes, but also their indirect interactions through all the other genes in the network. The topological overlap matrix was defined as *TOM_ij_* = (Σ_u_ *a_iu_a_uj_* + *a_ij_*)/(min (*k_i_*,*k_j_*) + 1 − *a_ij_*), where *k_i_* = Σ_u_ *a_iu_* was the node connectivity [18,19]. The expression 1 − *TOM_ij_* was used as a distance matrix in the hierarchical clustering of the transcript units to allow module detection [19].

The module’s eigengene was the first principal component of the matrix of expression values of a given module, which was adopted to characterize the gene expression profile.

### 2.3. Gene Functional Enrichment Analysis

Two enrichment methods were employed. For the over-representation analysis (ORA), the database for annotation, visualization and integrated discovery (DAVID, v6.7) (http://david.abcc.ncifcrf.gov/) was used to test the enrichment in gene sets with Gene Ontology (GO) and Kyoto Encyclopedia of Genes and Genomes (KEGG) pathway database terms, compared with the background list of all genes [20]. For the pathway topology(PT)-based analysis, Mirna enrIched paTHway Impact anaLysis (MITHrIL) was used [21,22].

### 2.4. Hub Gene Analysis

Hub genes are highly connected nodes. Unlike the protein–protein interaction network [23], WGCNA defines a whole network connectivity measure (*k_Total_*) for each gene based on its Pearson correlation coefficient with all of the other genes. Intra-module hubs can be determined by an intra-modular connectivity measure (*k_Within_*) to assess the connection strength of each individual gene compared to other genes in the same module. Intra-modular hub genes exhibit higher biologically significance compared with other genes, as intra-hub genes of a gene co-expression module may serve as candidate biomarkers and therapeutic targets in corresponding gene pathways.

## 3. Results

### 3.1. Differentially Expressed Genes

**Figure 1:**
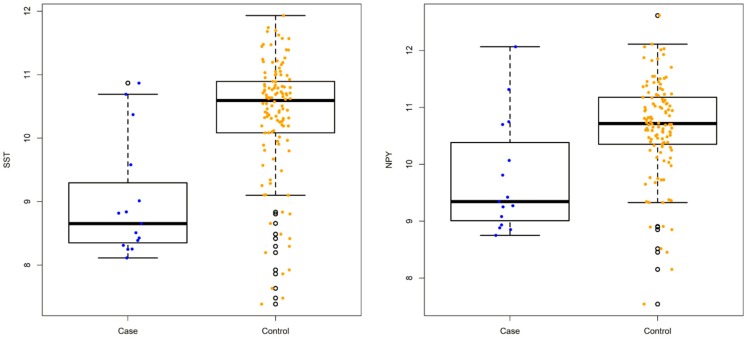
The expression levels of *SST* and *NPY* between cases and controls. The x-axis shows the group of cases (blue) and controls (yellow), and the y-axis shows gene expression levels (logFC).

A total of 678 differentially expressed genes were identified by LIMMA, including 379 up-regulated genes and 299 down-regulated genes (see Figure S2 and Table S2). In addition, multiple candidate genes implicated in schizophrenia or other psychiatric disorders were detected (see Figure 1 and Figure S3), such as *SST*, *NPY*, *SLC32A1*, *HINT1*, *RELN*, and *IFITM3* [24,25,26,27,28]. The gene functional enrichment analysis (over-representation analysis, ORA) showed that the up-regulated genes were significantly enriched in gene pathways such as the “MAPK signaling pathway” (KEGG: hsa04010) and “focal adhesions” (KEGG: hsa04510) (see Table S3). The enrichment analysis of the down-regulated genes showed that genes involved in the “mitochondrion” (GO: 0005739), “oxidative phosphorylation” (KEGG: hsa00190), and “proteasome” (KEGG: hsa03050) were significantly overrepresented. Furthermore, the expression of pathways associated with neurodegenerative diseases, such as Parkinson’s disease (KEGG: hsa05012) and Huntington’s disease (KEGG: hsa05016), were also reduced in patients with EDs (see Table S3). We also performed PT-based enrichment analysis by MITHrIL. MITHrIL also identified that the “MAPK signaling pathway” (KEGG: hsa04010) and “focal adhesions” (KEGG: hsa04510) are significantly overexpressed, while pathways associated with Alzheimer’s disease (KEGG: hsa05010), Parkinson’s disease (KEGG: hsa05012), and Huntington’s disease (KEGG: hsa05016) are down-regulated. Interestingly, MITHrIL additionally identified suppressed pathways involved in neurotransmission such as the “neurotrophin signaling pathway” (KEGG: hsa04722) and “GABAergic synapse” (KEGG: hsa04727). Taken together, the results generated by two enrichment methods are highly consistent (See Table S4).

### 3.2. Detection of Functional Modules in Gene Co-Expression Networks

**Figure 2:**
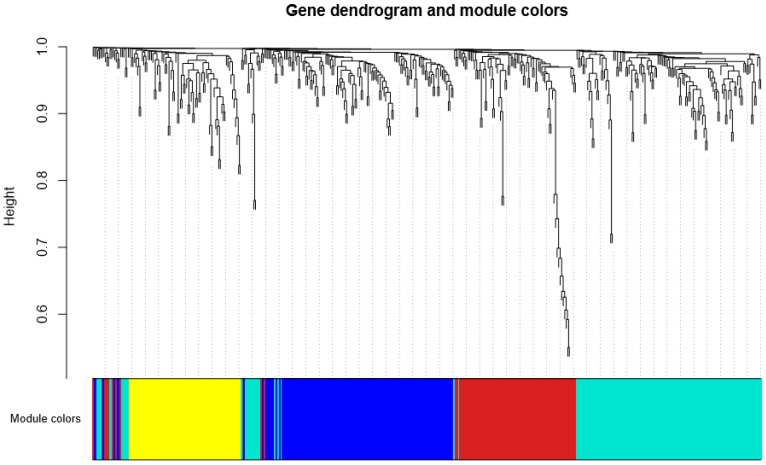
The gene dendrogram and the corresponding module colors. The upper section is the dendrogram of differentially expressed genes, a hierarchical cluster tree showing co-expression modules identified by weighted gene co-expression network analysis (WGCNA). Each leaf in the tree is one gene. The lower section indicates the modules of co-expressed genes, each color represents a different module and each module contains genes with similar expression patterns.

We further analyzed the 678 differentially expressed genes with WGCNA, a method of analysis which has been widely applied to the construction of the gene co-expression network and module detection. A gene co-expression network consists of gene expression profiles represented as nodes and gene connections, which occur if two genes are significantly co-expressed (determined by pairwise gene expression correlations). WGCNA first transforms the similarity matrix of the 678 genes containing co-expression similarities into the adjacency matrix containing connection strengths. The adjacency matrix is next transformed into a TOM, which captured not only the direct interaction between two genes, but also their indirect interactions through all the other genes in the network. Based on the TOM, WGCNA employs an unsupervised method to cluster the genes into modules. Four co-expression modules were identified. Topological properties of the detected modules are summarized in Table S5; functional and pathway enrichment analyses were performed for the genes in each module. Two modules were significantly enriched in genes with specific pathway roles (Figure 2 and Table S6; yellow module: 73 genes, including “mitochondrion” (GO: 0005739) and “oxidative phosphorylation” (KEGG: hsa00190); and turquoise module: 147 genes, including “neuropeptide hormone activity” (GO: 0005184) and “proteasome complex” (GO:0000502)). In parallel, we also performed PT-based enrichment analysis by MITHrI. MITHrI additionally identified both blue and brown modules associated with the important neurotransmission pathway “glutamatergic synapse” (KEGG: hsa04724), suggesting the advantage of using the PT-based method in functional enrichment analysis (See Table S7).

**Figure 3:**
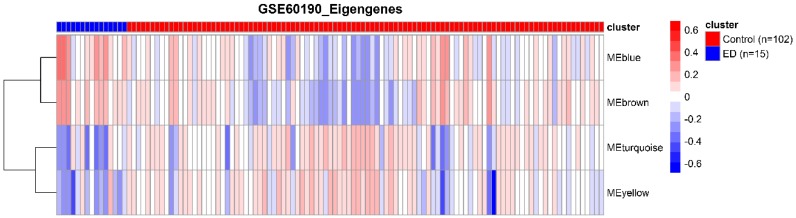
Heatmap of eigengenes. x-axis shows the cluster of cases (blue) and controls (red), y-axis shows the dendrogram of identified modules.

The eigengene was computed to represent the overall expression profiles of the genes in a certain module. The gene expression levels of module yellow and module turquoise were low in individuals with EDs, which suggests a high consistency between the pathways analysis and the co-expression-based gene network analysis (see Figure 3).

### 3.3. Identification of Hub Genes

According to network topology, hub genes with a high number of connections are considered to play critical roles in organizing the structure of biological networks. An intra-modular hub gene is defined as a gene with high connections within a module. Intra-modular hub genes tend to have high biological relevance.

We further investigated whether the intra-modular hub genes of a co-expression module were related to its enriched pathways. Additionally, the intra-module hub gene from a yellow module with the highest value of connectivity was a schizophrenia-associated gene known as *HINT1*, and two of the top five connected intra-module hubs in module yellow were *MRPS18C* and *NDUFA12*, which are implicated in the function of mitochondria. This suggests that the hub genes in the yellow module could influence the pathogenesis of EDs through mitochondria-related pathways (see Table S8).

### 3.4. Protein-Protein Interaction Network Analysis of Detected Modules

To further explore the direct interactions (protein–protein interaction) among genes of each detected module, we mapped the gene members of each module to the proteomic network in GeneMANIA [23,29]. In the yellow module, 21 genes are highly connected, forming the largest connected component (LCC). The LCC includes *HINT1*, eight genes encoding the subunits of NADH: ubiquinone oxidoreductase, and four genes encoding the subunits of ATP synthase. This result suggests the yellow module is highly associated with the function of NADH, ubiquinone oxidoreductase, and ATP synthase, which is consistent with results from WGCNA. In the turquoise module, 63 closely connected genes form the LCC, including ten genes encoding subunits of proteasome. This observation is also consistent with results from WGCNA (Figure S4).

## 4. Discussion

In this study, we analyzed the ED-related gene expression data generated by the study of Jaffe et al. [14]. Specifically, Jaffe et al. identified only six differentially expressed genes between EDs and controls using a surrogate variable analysis. However, complex diseases such as EDs are unlikely driven by a few gene targets. More genes underlying EDs may be detected by optimized statistical methods. Here, we used an empirical Bayesian method, LIMMA, to identify the differentially expressed genes between diseased individuals with ED and diseased controls. In this analysis, we identified a total of 678 differentially expressed candidate genes in EDs. Gene enrichment analysis and co-expression network analysis further revealed dysregulated gene expression in EDs, which has previously been implicated in other mental disorders or pathways associated with oxidative phosphorylation.

The LIMMA analysis first detected a number of down-regulated genes in individuals with EDs that are known to be suppressed in schizophrenia or major depressive disorder, such as *SST*, *NPY*, *SLC32A1*, *HINT1*, and *RELN* [24,25,26,28]. Both *SST* and *NPY* are neuropeptide genes. The products of *SST* and *NPY*, somatostatin and neuropeptide Y, respectively, often serve as important biomarkers in psychiatric disorders [30]. Moreover, neuropeptide Y is one the most potent orexigenic peptides found in the brain. It stimulates food intake with a preferential effect on carbohydrate intake. It decreases latency in eating, increases motivation to eat, and delays satiety by augmenting meal size [31]. Furthermore, overexpression of the immune system-associated gene *IFITM3*, was found in individuals with EDs (see Figure S2), and has also been repeatedly reported to be up-regulated in studies of schizophrenia and autism [27,32]. Thus, our results indicate that similar biological mechanisms may be involved in the development of EDs and other psychiatric disorders. Indeed, comorbidity of AN and other psychiatry disorders is common, such as obsessive-compulsive disorders, depression, anxiety disorders and alcoholism.

In addition, the gene functional enrichment analysis indicated that the gene pathways related to mitochondrial functioning and the oxidative phosphorylation process are significantly suppressed in EDs. Altered mitochondrial functioning and oxidative stress have been found in the leukocytes of patients with AN [33]. The association between mitochondrial dysfunction and disturbed feeding behaviors has also been demonstrated in a mouse model [34].

Considering that psychiatric disorders are complicated and heterogeneous, using WGCNA we further identified a gene co-expression module which is highly associated with mitochondrial functioning and oxidative phosphorylation. The results of our study together with existing research suggests that mitochondrial functioning and oxidative phosphorylation may play key roles in EDs.

The major limitations of this study were the relatively small sample size of ED cases and the lack of replication cohorts. The second limitation is combining different ED subtypes as a group. AN and BN are two major types of EDs. People with AN see themselves as overweight even though they are dangerously thin from starving themselves. People with BN eat unusually large amounts of food (binge eat) and then compensate by purging (vomiting, or taking laxatives or diuretics), fasting, or excessive exercise. Although twin studies in AN and BN eating disorders found a moderate overlapping of both genetic and unique environmental factors that influence the two conditions [35,36], each type of ED may still be associated with a set of subtype-specific genes. In this study, since most participants only have been diagnosed as binge eating, the top differentially expressed genes detected in our analysis may be generally dysregulated in both BN and AN, or specifically dysregulated in BN. Genes specifically dysregulated in AN may be omitted. We anticipate future studies with greater sample sizes of each ED subtype will be able to better uncover the different biological mechanisms underlying different ED subtypes. The third limitation is a large difference of sex ratio between cases and controls. The differentially expressed genes in brain DLPFC tissues between males and females might also influence our results. This issue can also be solved by collecting more samples in future studies. It is also worth mentioning that the current results cannot rule out the effects of comorbid depression in patients with ED. Indeed, most patients with EDs (>90%) evidenced comorbid mood disorders, largely depression [37]. However, depression is a very common mental disorder [38], while EDs are relatively rare [39]. Depression has high comorbidity with other chronic or physical diseases, such as cancer, heart disease, and diabetes [40,41]. Moreover, in addition to genetic factors, depression may be triggered by risk factors such as illness, poor nutrition, hormonal imbalance, stress, and anxiety. Some of those risk factors mentioned above are also associated with EDs. Taken together, we believe our results mainly reflect the dysregulated pathways associated with EDs, while could be affected by comorbidities.

In summary, our results suggested that shared gene pathways and brain circuitries may underlie EDs and various psychiatric disorders. Alteration of those gene pathways may be associated with the psychiatric syndromes of EDs. Moreover, our results also indicate that mitochondrial functioning and the oxidative phosphorylation process may be specific in the pathology of EDs, and decreased activity of mitochondrial functioning and the oxidative phosphorylation process may cause predisposition to the development of EDs. Future functional studies or genomic studies with a large number of ED cases will provide more insight into the genomic underpinnings between EDs and mitochondria.

## Supplementary Materials

The following are available online at http://www.mdpi.com/2073-4425/9/7/325/s1, Figure S1: The quantities in the chosen columns vs. the soft threshold power, Figure S2: Heat map of differentially expressed genes. X-axis shows the cluster of cases (cluster 1) and controls (cluster 2), Y-axis shows the dendrogram of differentially expressed genes, Figure S3: a. Expression level of *SLC32A1* between cases and controls, b. Expression level of *HINT1* between cases and controls, c. Expression level of *IFITM3* between cases and controls, d. Expression level of *RELN* between cases and controls, Figure S4: Protein-Protein Interaction Network structure of detected modules, a. Module yellow, b. Module turquoise, c. Module blue, d. Module brown, Table S1: Sample demographics; Table S2: Differentially expressed genes; Table S3: Functional enrichment of differentially expressed genes; Table S4: Functional enrichment of differentially expressed genes (MITHrIL); Table S5: Topological properties of the detected modules by WGCNA; Table S6: Functional enrichment of the WGCNA Modules., Table S7: Functional enrichment of the WGCNA Modules (MITHrIL), Table S8: Connectivity strength of intra-hub genes in the functional enriched modules. For module turquoise and blue (>100 genes), the top 10 connected genes (*k_within_*) were listed. For module brown and yellow (<100 genes), the top 5 connected genes (*k_within_*) were listed.
