# Supplementary material for: Weighted Gene Co-Expression Network Analysis Reveals Dysregulation of Mitochondrial Oxidative Phosphorylation in Eating Disorders
Source: Genes (Basel). 2018 Jun 28;9(7):325. doi: 10.3390/genes9070325 (PMC6070803; doi:10.3390/genes9070325)
Supplement: Supplementary file 1 [file genes-09-00325-s001.pdf]

Figure. S1 The quantities in the chosen columns vs. the soft threshold power

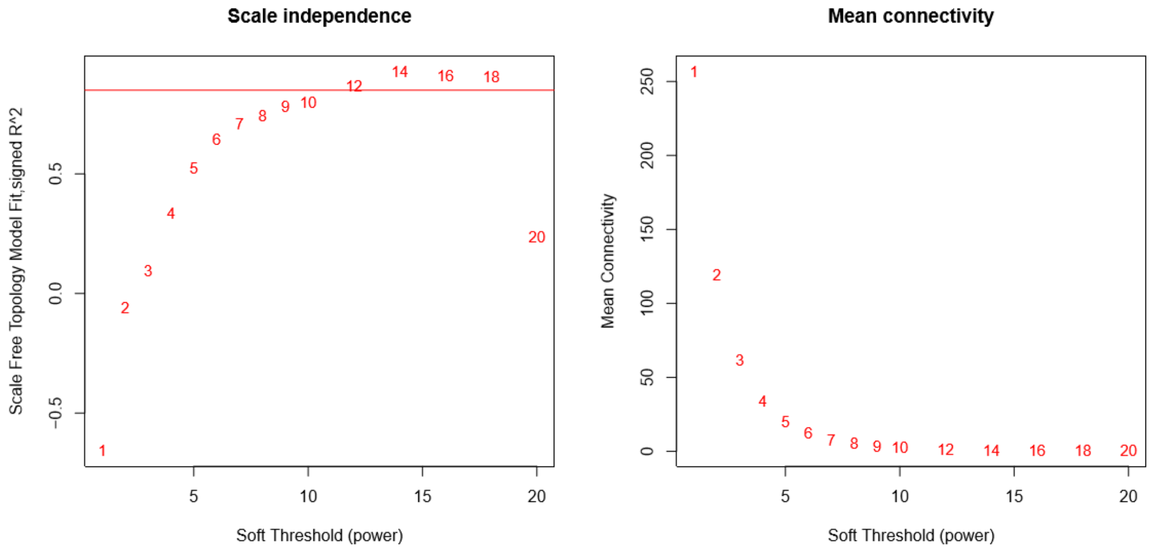

Figure S2. Heat map of differentially expressed genes. X-axis shows the cluster of cases (cluster 1) and controls (cluster 2), Y-axis shows the dendrogram of differentially expressed genes.

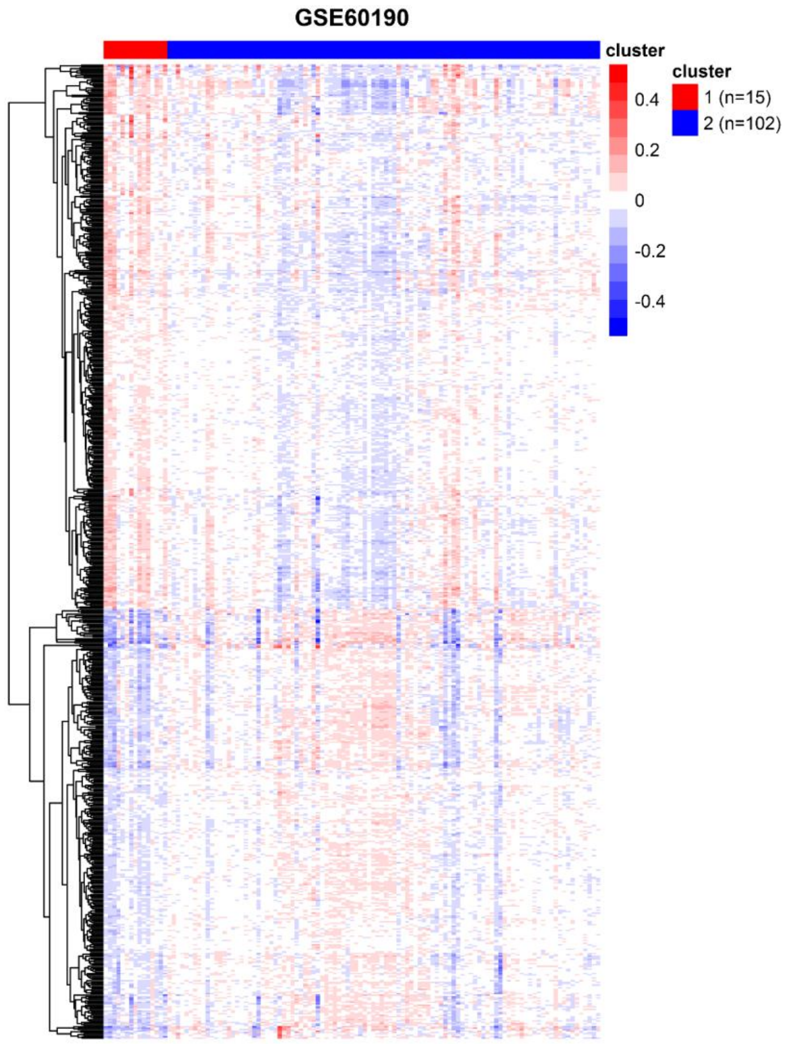

Figure. S3

a. Expression level of *SLC32A1* between cases and controls

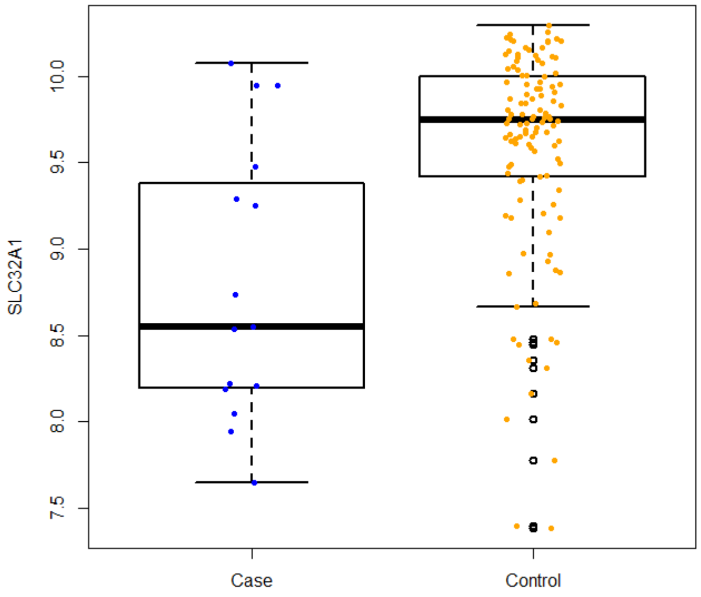

b. Expression level of *HINT1* between cases and controls

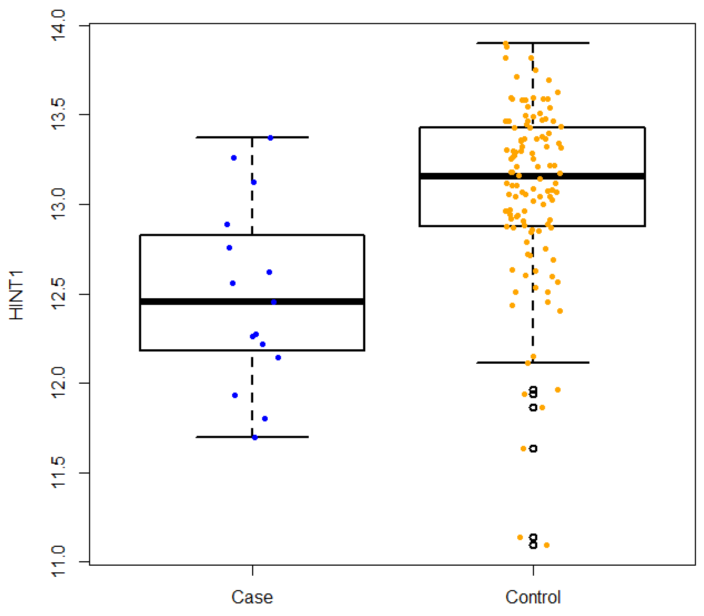

c. Expression level of *IFITM3* between cases and controls

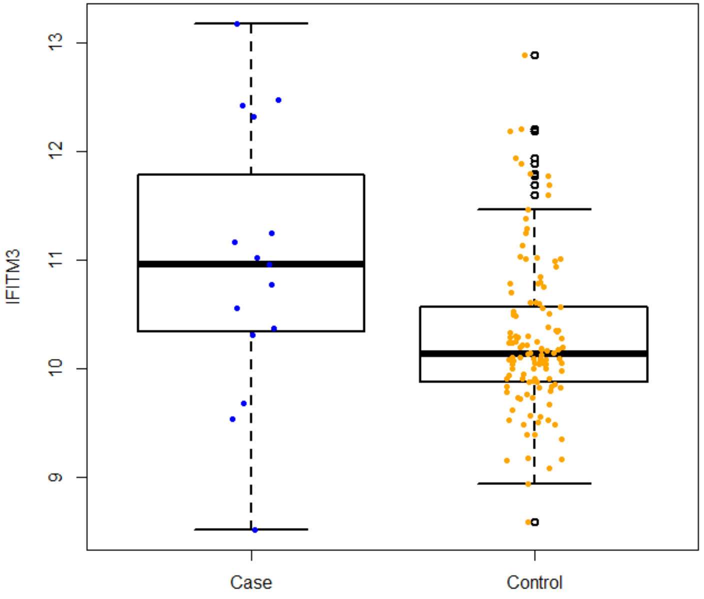

d. Expression level of *RELN* between cases and controls

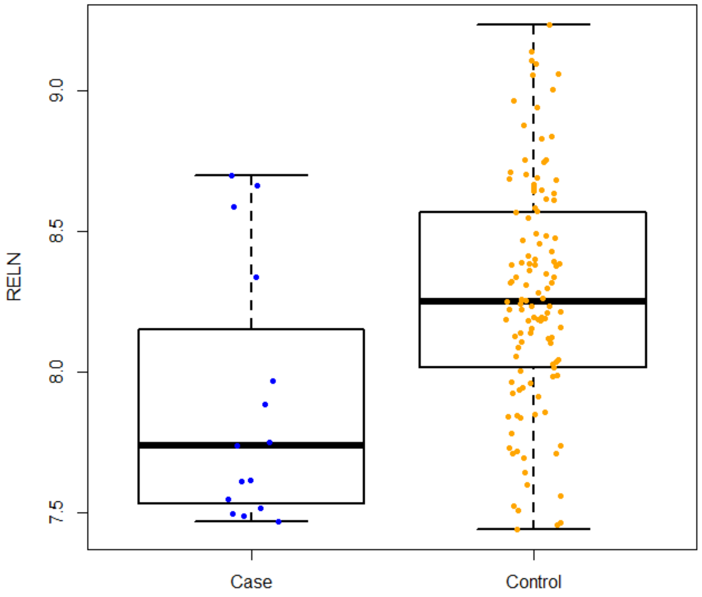

Figure. S4 Protein-Protein Interaction Network structure of detected modules

a. Module yellow

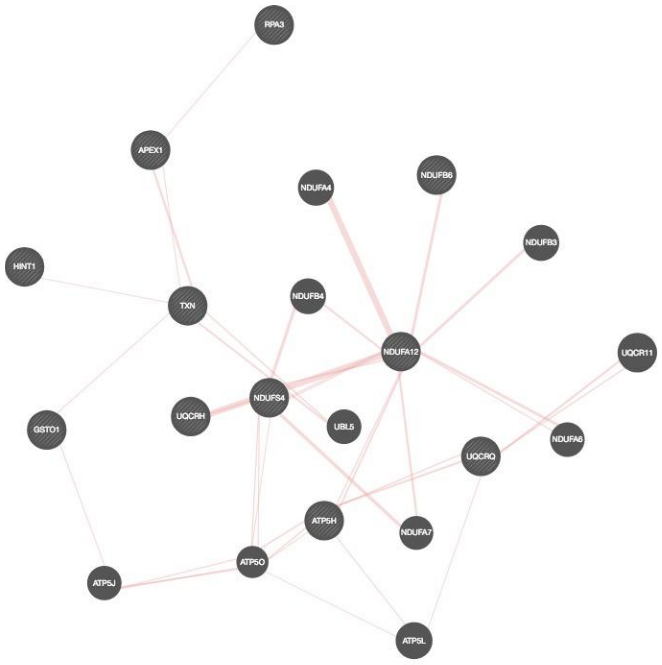

b. Module turquoise

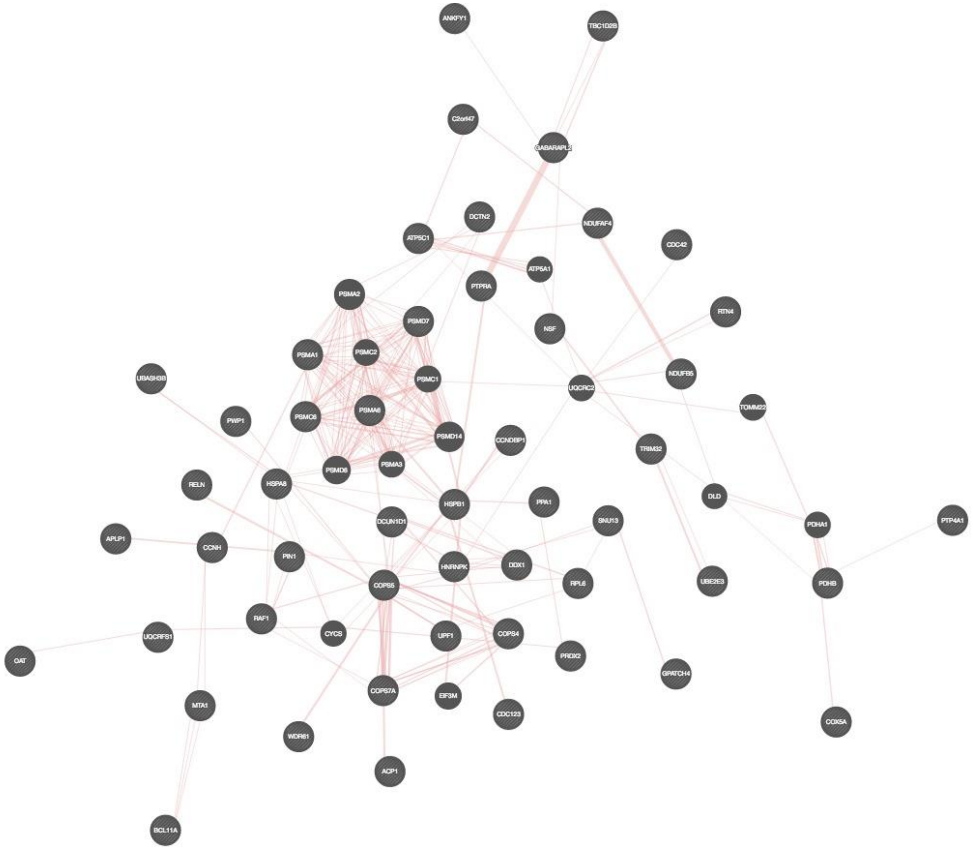

c. Module blue

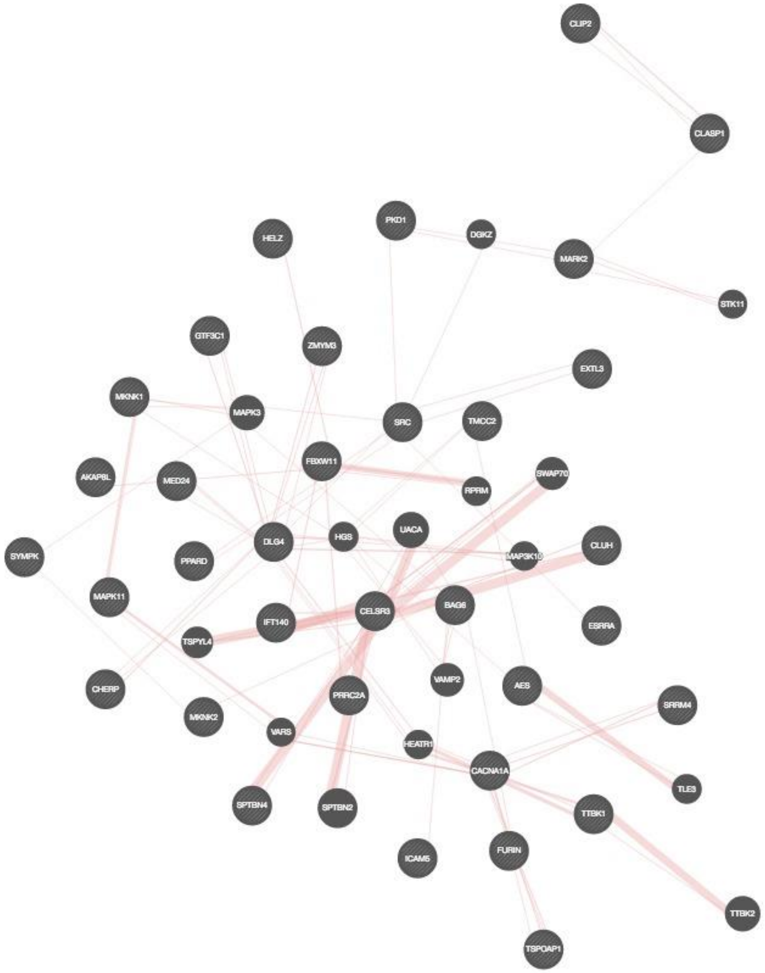

d. Module brown

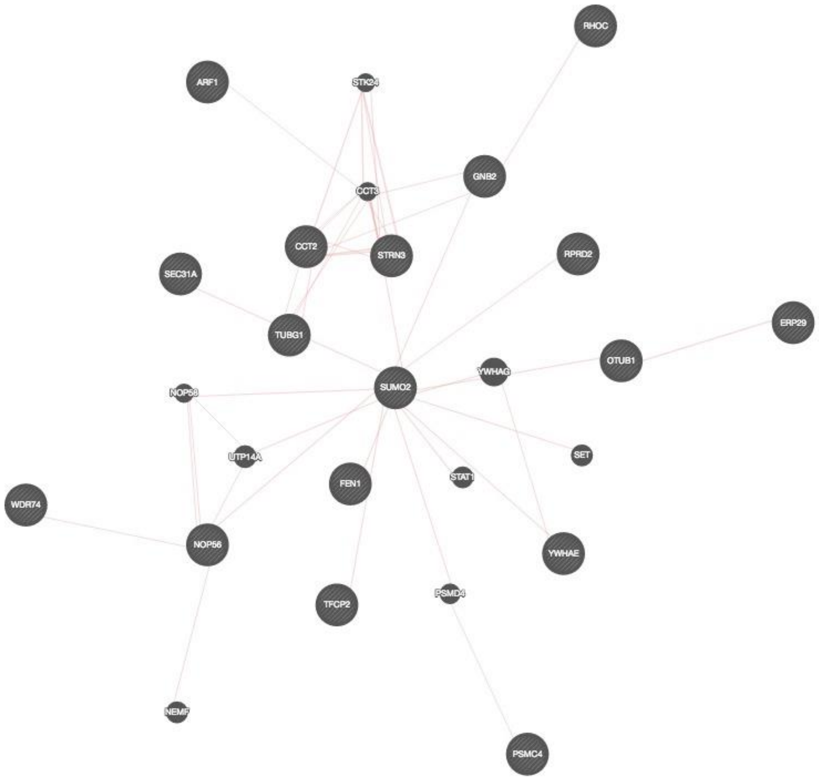

Table S1. Sample demographics

	N	Sex (% F)	Age	pH	PMI	RIN	Subtype	MoodDx	Substance Dx	Suicide	Antidepr Tox
ED	15	86.7	40.1 ±8.1	6.2 ±0.2	32.3 ±19.5	7.57 ±0.88	7 BN<br>2 AN-BN<br>3 AN<br>3 NOS	86.70%	53.30%	73.30%	66.70%
Control	102	20.5	42.5 ±17.2	6.6±0.3	27.2±14.8	8.3±0.69					

Abbreviations: AN, anorexia nervosa (restricting); AN-BN, anorexia nervosa, binge–purge type; AntidepressTox, percentage of cases on antidepressants at the time of death as measured by blood/brain toxicology; BN, bulimia nervosa; ED, eating disorder; MoodDx, presence of comorbid Axis I mood disorder diagnosis; NOS, eating disorder not otherwise specified; pH, brain pH; PMI, postmortem interval in hours; RIN, RNA integrity number; Substance Dx, presence of comorbid Axis I substance use disorder. All numerical cells are mean±s.d.

Table S2. Differentially expressed genes

Expression	genname	logFC	AveExpr	t	*P*.Value	adj.*P*.Val	B
Up-regulation	*TNFRSF6B*	0.81	8.01	6.15	1.07E-08	1.42E-04	9.47
	TSHZ2	0.47	8.69	5.44	2.84E-07	9.35E-04	6.48
	AP1G2	0.45	8.50	5.16	1.01E-06	1.37E-03	5.33
	HYOU1	0.49	9.08	5.15	1.05E-06	1.37E-03	5.29
	ELK1	0.53	9.76	5.13	1.14E-06	1.37E-03	5.21
	CHTF18	0.27	7.74	5.11	1.23E-06	1.37E-03	5.15
	IGSF9B	0.41	8.00	5.07	1.49E-06	1.52E-03	4.97
	COL4A1	0.59	8.28	5.02	1.80E-06	1.58E-03	4.80
	SIN3B	0.51	8.86	4.78	4.97E-06	2.58E-03	3.88
	PPP1R10	0.38	8.40	4.77	5.21E-06	2.58E-03	3.83
	ULK3	0.35	8.11	4.77	5.26E-06	2.58E-03	3.83
	DGCR2	0.47	9.63	4.71	6.78E-06	3.21E-03	3.60
	ABCA7	0.37	7.91	4.67	7.84E-06	3.26E-03	3.46
	TTC14	0.46	9.21	4.67	8.02E-06	3.26E-03	3.44
	LOC146177	0.26	7.76	4.66	8.15E-06	3.26E-03	3.43
	ST6GALNAC6	0.58	10.51	4.55	1.31E-05	4.69E-03	3.00
	PTCHD1	0.34	8.69	4.51	1.56E-05	5.27E-03	2.84
	SRC	0.40	9.07	4.49	1.64E-05	5.28E-03	2.80
	PARC	0.33	8.07	4.44	2.03E-05	5.78E-03	2.61
	CSDA	0.78	8.90	4.44	2.06E-05	5.78E-03	2.59
	SFRS14	0.34	8.30	4.42	2.20E-05	5.78E-03	2.53
	LOC654342	0.34	7.85	4.42	2.23E-05	5.78E-03	2.52
	CHMP1A	0.40	9.21	4.37	2.64E-05	6.36E-03	2.37
	HS.490981	0.40	8.38	4.37	2.65E-05	6.36E-03	2.36
	DGKG	0.27	7.80	4.36	2.73E-05	6.36E-03	2.34
	LAMC1	0.33	8.99	4.36	2.77E-05	6.36E-03	2.33
	MTHFR	0.29	7.93	4.36	2.79E-05	6.36E-03	2.32
	ZNF562	0.27	8.59	4.33	3.12E-05	6.89E-03	2.22
	CACNA1A	0.55	8.29	4.33	3.13E-05	6.89E-03	2.22
	GADD45B	0.56	7.92	4.29	3.65E-05	7.81E-03	2.08
	HELZ	0.30	8.96	4.29	3.71E-05	7.81E-03	2.06

	CHERP	0.22	8.03	4.29	3.72E-05	7.81E-03	2.06
	LOC644931	0.51	8.44	4.27	4.03E-05	8.07E-03	1.99
	SHC1	0.42	9.14	4.26	4.13E-05	8.07E-03	1.97
	HS.388347	0.32	8.26	4.26	4.19E-05	8.07E-03	1.95
	IFT140	0.26	7.85	4.25	4.21E-05	8.07E-03	1.95
	CACNA1C	0.26	8.04	4.22	4.82E-05	8.52E-03	1.83
	ZER1	0.38	8.73	4.21	4.92E-05	8.52E-03	1.81
	GNAO1	0.63	11.80	4.21	5.05E-05	8.52E-03	1.78
	ADCY6	0.25	8.03	4.20	5.24E-05	8.52E-03	1.75
	NPIP	0.47	10.30	4.19	5.35E-05	8.52E-03	1.73
	RNF40	0.27	8.56	4.16	6.09E-05	9.37E-03	1.62
	ANKRD20A1	0.62	10.26	4.15	6.19E-05	9.42E-03	1.60
	BAG4	0.35	8.07	4.11	7.20E-05	1.05E-02	1.47
	EXTL3	0.44	9.57	4.08	8.30E-05	1.16E-02	1.34
	PHYHIP	0.57	9.27	4.08	8.34E-05	1.16E-02	1.33
	RGS11	0.32	8.07	4.05	9.01E-05	1.23E-02	1.26
	GNB2	0.27	7.94	4.05	9.17E-05	1.24E-02	1.25
	TUBGCP6	0.20	7.77	4.05	9.25E-05	1.24E-02	1.24
	SLC38A11	0.34	8.28	4.03	9.88E-05	1.27E-02	1.18
	OGDH	0.44	9.07	4.02	1.04E-04	1.29E-02	1.14
	FURIN	0.18	7.75	4.01	1.05E-04	1.29E-02	1.13
	NARFL	0.27	8.30	4.00	1.11E-04	1.33E-02	1.07
	ADAM11	0.41	8.99	3.99	1.14E-04	1.35E-02	1.05
	HS.34558	0.26	8.20	3.99	1.15E-04	1.35E-02	1.04
	CEBPD	0.66	9.28	3.98	1.19E-04	1.35E-02	1.02
	HS.181500	0.31	8.32	3.98	1.20E-04	1.35E-02	1.00
	MT1M	0.86	9.40	3.98	1.21E-04	1.35E-02	1.00
	RSAD1	0.27	7.90	3.97	1.22E-04	1.35E-02	0.99
	CELSR3	0.38	9.92	3.97	1.23E-04	1.35E-02	0.99
	BZRAP1	0.44	9.50	3.97	1.24E-04	1.35E-02	0.98
	LOC729008	0.23	7.71	3.97	1.25E-04	1.35E-02	0.97
	ST5	0.26	8.01	3.96	1.27E-04	1.36E-02	0.96
	SLC9A5	0.26	7.91	3.95	1.34E-04	1.37E-02	0.91

	COLQ	0.21	8.06	3.95	1.35E-04	1.37E-02	0.90
	MBOAT7	0.45	9.48	3.94	1.36E-04	1.37E-02	0.90
	SMARCD3	0.27	8.51	3.94	1.36E-04	1.37E-02	0.90
	B4GALNT4	0.47	8.95	3.94	1.37E-04	1.38E-02	0.89
	ANKFY1	0.21	8.07	3.94	1.39E-04	1.38E-02	0.88
	CIC	0.28	8.36	3.94	1.40E-04	1.38E-02	0.87
	LOC728734	0.57	10.83	3.93	1.43E-04	1.39E-02	0.85
	TRIM52	0.23	8.12	3.91	1.51E-04	1.44E-02	0.80
	ADAMTS9	0.34	7.78	3.91	1.53E-04	1.44E-02	0.79
	PKD1	0.59	9.32	3.91	1.55E-04	1.44E-02	0.78
	MBTPS1	0.33	8.16	3.91	1.55E-04	1.44E-02	0.78
	SOHLH1	0.43	8.42	3.90	1.57E-04	1.44E-02	0.77
	RIMS4	0.43	8.77	3.90	1.59E-04	1.45E-02	0.76
	LPPR2	0.41	8.92	3.90	1.60E-04	1.45E-02	0.75
	RAD9A	0.22	7.97	3.90	1.63E-04	1.46E-02	0.74
	RFNG	0.42	9.60	3.89	1.64E-04	1.46E-02	0.73
	PPP2R5B	0.27	9.40	3.89	1.66E-04	1.47E-02	0.72
	SYMPK	0.39	8.14	3.88	1.72E-04	1.50E-02	0.68
	ANGPT2	0.37	7.82	3.85	1.89E-04	1.61E-02	0.60
	C19ORF28	0.21	7.92	3.84	1.97E-04	1.65E-02	0.57
	CCDC109A	0.21	8.04	3.84	2.01E-04	1.67E-02	0.55
	SERPING1	0.29	7.94	3.84	2.02E-04	1.67E-02	0.54
	USP35	0.19	7.75	3.83	2.07E-04	1.70E-02	0.52
	UNKL	0.30	8.12	3.82	2.13E-04	1.74E-02	0.49
	LOC441268	0.37	8.82	3.82	2.14E-04	1.74E-02	0.49
	PTP4A2	0.37	8.85	3.81	2.18E-04	1.75E-02	0.48
	FLJ33534	0.21	7.73	3.81	2.18E-04	1.75E-02	0.48
	BTBD9	0.30	7.95	3.81	2.24E-04	1.77E-02	0.45
	ADCY6	0.29	8.67	3.80	2.30E-04	1.79E-02	0.43
	TUFT1	0.24	8.15	3.80	2.31E-04	1.79E-02	0.42
	ZFX	0.16	7.69	3.79	2.34E-04	1.80E-02	0.41
	DDEFL1	0.30	8.49	3.79	2.41E-04	1.82E-02	0.38
	UPF1	0.32	8.56	3.79	2.41E-04	1.82E-02	0.38

	CLASP1	0.33	10.32	3.78	2.45E-04	1.82E-02	0.37
	YTHDF1	0.23	9.54	3.78	2.46E-04	1.82E-02	0.37
	GCAT	0.33	8.73	3.78	2.48E-04	1.82E-02	0.36
	LOC650580	0.33	7.82	3.78	2.50E-04	1.82E-02	0.35
	PAPD5	0.39	8.76	3.77	2.52E-04	1.82E-02	0.35
	MLL	0.21	8.28	3.77	2.55E-04	1.82E-02	0.33
	LOC440359	0.41	7.93	3.76	2.60E-04	1.83E-02	0.32
	TAOK2	0.38	8.23	3.75	2.70E-04	1.86E-02	0.28
	TBC1D3G	0.23	7.90	3.75	2.72E-04	1.86E-02	0.28
	TEF	0.46	9.72	3.75	2.77E-04	1.89E-02	0.26
	SLC39A14	0.25	8.17	3.74	2.80E-04	1.89E-02	0.25
	PTPRA	0.40	9.87	3.74	2.81E-04	1.89E-02	0.25
	DLG4	0.72	11.79	3.74	2.85E-04	1.90E-02	0.23
	COL6A1	0.41	8.61	3.73	2.91E-04	1.91E-02	0.22
	TTC14	0.34	9.29	3.73	2.92E-04	1.91E-02	0.21
	EDN1	0.44	7.95	3.73	2.96E-04	1.92E-02	0.20
	MAPK11	0.38	8.41	3.72	3.08E-04	1.94E-02	0.17
	COL11A1	0.30	8.27	3.72	3.10E-04	1.94E-02	0.16
	LOC728452	0.25	7.75	3.72	3.11E-04	1.94E-02	0.16
	IL18BP	0.27	7.98	3.71	3.12E-04	1.94E-02	0.15
	RORB	0.50	9.14	3.71	3.16E-04	1.94E-02	0.14
	CLIP2	0.42	9.71	3.71	3.17E-04	1.94E-02	0.14
	DPP9	0.38	8.58	3.70	3.25E-04	1.95E-02	0.12
	CHD8	0.40	9.67	3.70	3.31E-04	1.97E-02	0.10
	HDAC5	0.30	8.42	3.70	3.31E-04	1.97E-02	0.10
	NRBP1	0.21	7.86	3.70	3.33E-04	1.97E-02	0.10
	FLJ37078	0.42	8.43	3.70	3.33E-04	1.97E-02	0.10
	RPIA	0.23	8.12	3.69	3.37E-04	1.97E-02	0.09
	FGF17	0.29	7.75	3.69	3.40E-04	1.97E-02	0.08
	YWHAE	0.56	8.98	3.69	3.40E-04	1.97E-02	0.08
	LOC613037	0.61	11.76	3.69	3.41E-04	1.97E-02	0.08
	RANBP10	0.19	8.19	3.69	3.45E-04	1.98E-02	0.07
	PBX1	0.17	7.72	3.68	3.55E-04	2.00E-02	0.04

	SCARA3	0.28	7.85	3.68	3.58E-04	2.00E-02	0.03
	SYT7	0.48	10.48	3.67	3.59E-04	2.00E-02	0.03
	MKL1	0.23	8.38	3.67	3.60E-04	2.00E-02	0.03
	GADD45A	0.40	9.29	3.67	3.66E-04	2.01E-02	0.01
	KLHL18	0.20	7.96	3.67	3.66E-04	2.01E-02	0.01
	NAT9	0.20	8.76	3.67	3.66E-04	2.01E-02	0.01
	NOL5A	0.36	9.96	3.66	3.71E-04	2.01E-02	0.00
	TNFRSF25	0.56	9.30	3.66	3.72E-04	2.01E-02	0.00
	KIAA0460	0.24	7.93	3.66	3.80E-04	2.03E-02	-0.02
	AKAP8L	0.42	8.74	3.66	3.81E-04	2.03E-02	-0.02
	MEG3	0.49	11.11	3.66	3.83E-04	2.04E-02	-0.03
	ZBTB16	0.40	9.01	3.65	3.86E-04	2.05E-02	-0.04
	GRB2	0.24	8.67	3.65	3.94E-04	2.08E-02	-0.05
	BAT2	0.42	9.35	3.64	4.00E-04	2.08E-02	-0.07
	KIAA0664	0.36	8.66	3.63	4.12E-04	2.13E-02	-0.09
	TAOK1	0.37	8.33	3.63	4.15E-04	2.14E-02	-0.10
	SS18L1	0.27	8.91	3.63	4.18E-04	2.14E-02	-0.11
	ATG16L2	0.23	8.06	3.63	4.23E-04	2.14E-02	-0.11
	LIMK1	0.30	8.10	3.62	4.36E-04	2.18E-02	-0.14
	GOLGA8A	0.48	8.15	3.62	4.37E-04	2.18E-02	-0.14
	RN7SK	0.49	8.04	3.62	4.41E-04	2.18E-02	-0.15
	ICAM2	0.40	8.83	3.60	4.58E-04	2.23E-02	-0.19
	MEIS3	0.27	7.95	3.60	4.61E-04	2.23E-02	-0.19
	STK38	0.25	8.41	3.60	4.70E-04	2.25E-02	-0.21
	MFHAS1	0.22	7.83	3.59	4.82E-04	2.30E-02	-0.23
	TYW3	0.27	8.35	3.59	4.86E-04	2.31E-02	-0.24
	LOC91461	0.26	8.22	3.58	4.91E-04	2.32E-02	-0.25
	ATXN7L3	0.45	10.58	3.58	5.07E-04	2.35E-02	-0.28
	MKNK1	0.29	8.81	3.57	5.08E-04	2.35E-02	-0.28
	NUPR1	0.32	8.31	3.57	5.14E-04	2.36E-02	-0.29
	FLJ25006	0.28	8.58	3.57	5.14E-04	2.36E-02	-0.29
	COL23A1	0.18	7.85	3.56	5.25E-04	2.40E-02	-0.31
	APLP1	0.63	10.04	3.56	5.26E-04	2.40E-02	-0.31

	ST5	0.29	8.04	3.56	5.30E-04	2.40E-02	-0.32
	TMEM16F	0.21	8.00	3.56	5.31E-04	2.40E-02	-0.32
	ENDOGL1	0.43	8.23	3.55	5.44E-04	2.42E-02	-0.34
	LOC90113	0.36	8.34	3.55	5.61E-04	2.43E-02	-0.37
	LOC389634	0.20	7.83	3.54	5.63E-04	2.43E-02	-0.37
	STK35	0.25	8.00	3.54	5.76E-04	2.47E-02	-0.39
	IGHMBP2	0.16	7.74	3.54	5.80E-04	2.47E-02	-0.39
	KIF1B	0.31	8.17	3.54	5.80E-04	2.47E-02	-0.40
	C1ORF152	0.23	7.84	3.53	5.90E-04	2.49E-02	-0.41
	ARHGAP26	0.25	7.94	3.52	6.02E-04	2.52E-02	-0.43
	PROSAPIP1	0.41	10.19	3.52	6.09E-04	2.54E-02	-0.44
	HDHD1A	0.19	8.12	3.52	6.11E-04	2.54E-02	-0.44
	GCS1	0.18	7.80	3.52	6.12E-04	2.54E-02	-0.44
	MGAT4B	0.23	8.42	3.52	6.14E-04	2.54E-02	-0.45
	PVR	0.19	7.99	3.52	6.18E-04	2.54E-02	-0.45
	MLXIPL	0.21	8.10	3.52	6.18E-04	2.54E-02	-0.45
	ISLR2	0.56	9.48	3.52	6.21E-04	2.54E-02	-0.46
	PHF1	0.28	8.11	3.51	6.26E-04	2.55E-02	-0.46
	UBE2J2	0.17	7.86	3.51	6.36E-04	2.56E-02	-0.48
	HMBOX1	0.21	8.04	3.51	6.37E-04	2.56E-02	-0.48
	PPARD	0.21	8.00	3.51	6.41E-04	2.56E-02	-0.48
	DAPK3	0.37	8.27	3.51	6.42E-04	2.56E-02	-0.48
	STK36	0.36	9.72	3.50	6.47E-04	2.57E-02	-0.49
	AP2A2	0.39	9.39	3.50	6.48E-04	2.57E-02	-0.49
	TNPO1	0.33	8.88	3.50	6.62E-04	2.61E-02	-0.51
	SESN1	0.28	9.87	3.49	6.70E-04	2.62E-02	-0.52
	CORO2B	0.31	9.49	3.49	6.74E-04	2.63E-02	-0.53
	GTF3C1	0.35	8.82	3.49	6.80E-04	2.64E-02	-0.54
	CAMTA2	0.19	7.78	3.49	6.85E-04	2.65E-02	-0.54
	6-Mar	0.60	10.83	3.48	7.05E-04	2.71E-02	-0.57
	UCN	0.14	7.69	3.47	7.16E-04	2.72E-02	-0.58
	MARK2	0.33	8.42	3.47	7.29E-04	2.76E-02	-0.60
	LOC285636	0.27	9.28	3.47	7.33E-04	2.76E-02	-0.60

	SNPH	0.41	9.04	3.46	7.42E-04	2.78E-02	-0.61
	HS.538861	0.27	8.30	3.46	7.52E-04	2.79E-02	-0.63
	MTA1	0.27	9.12	3.45	7.72E-04	2.84E-02	-0.65
	CENTB5	0.26	7.92	3.45	7.82E-04	2.86E-02	-0.66
	ICAM5	0.34	8.34	3.45	7.84E-04	2.86E-02	-0.66
	DIAPH1	0.17	7.78	3.44	7.98E-04	2.90E-02	-0.68
	MKNK2	0.24	8.07	3.43	8.18E-04	2.95E-02	-0.70
	HS.584149	0.13	7.67	3.43	8.21E-04	2.95E-02	-0.70
	TMCC2	0.35	8.28	3.43	8.36E-04	2.96E-02	-0.72
	WDR74	0.43	9.08	3.43	8.42E-04	2.96E-02	-0.72
	TOP1MT	0.21	8.07	3.42	8.53E-04	2.96E-02	-0.74
	LUC7L	0.23	9.14	3.42	8.62E-04	2.97E-02	-0.75
	C14ORF130	0.14	7.68	3.42	8.64E-04	2.97E-02	-0.75
	DUSP8	0.58	9.47	3.42	8.65E-04	2.97E-02	-0.75
	UBE2H	0.41	8.46	3.41	8.92E-04	3.04E-02	-0.78
	SDF4	0.32	9.59	3.41	8.97E-04	3.04E-02	-0.78
	IGFBP4	0.29	7.95	3.40	9.03E-04	3.05E-02	-0.79
	HSPB1	0.68	10.57	3.40	9.08E-04	3.05E-02	-0.79
	MEIS3	0.31	8.57	3.40	9.17E-04	3.05E-02	-0.80
	SORCS1	0.31	8.40	3.40	9.19E-04	3.05E-02	-0.80
	GRIN1	0.28	8.09	3.40	9.21E-04	3.05E-02	-0.80
	PARVB	0.16	7.83	3.40	9.25E-04	3.05E-02	-0.81
	CALN1	0.30	9.44	3.40	9.29E-04	3.05E-02	-0.81
	KIAA1853	0.35	9.38	3.39	9.38E-04	3.06E-02	-0.82
	FKRP	0.27	8.56	3.39	9.45E-04	3.07E-02	-0.83
	DYNC2H1	0.22	7.99	3.39	9.58E-04	3.09E-02	-0.84
	NPIP	0.46	9.12	3.38	9.73E-04	3.12E-02	-0.85
	IQSEC2	0.36	9.04	3.38	9.79E-04	3.12E-02	-0.86
	SEC31A	0.33	9.58	3.38	9.83E-04	3.13E-02	-0.86
	CD276	0.18	8.06	3.38	9.93E-04	3.13E-02	-0.87
	BRD3	0.38	9.55	3.38	9.95E-04	3.13E-02	-0.87
	CYLN2	0.42	9.49	3.38	9.96E-04	3.13E-02	-0.87
	HS.473191	0.29	8.99	3.38	9.96E-04	3.13E-02	-0.87

	SORBS1	0.16	7.69	3.37	1.00E-03	3.13E-02	-0.88
	BAT2	0.32	8.75	3.37	1.00E-03	3.13E-02	-0.88
	GAA	0.21	7.84	3.37	1.02E-03	3.15E-02	-0.89
	RIMS2	0.37	8.81	3.36	1.03E-03	3.18E-02	-0.90
	LOC647389	0.45	9.16	3.36	1.05E-03	3.22E-02	-0.91
	RHOC	0.34	8.46	3.36	1.05E-03	3.23E-02	-0.92
	HS.193767	0.50	9.02	3.36	1.06E-03	3.25E-02	-0.93
	C12ORF53	0.38	8.27	3.35	1.10E-03	3.32E-02	-0.96
	BAT3	0.23	9.15	3.34	1.11E-03	3.34E-02	-0.96
	ZNF641	0.24	8.19	3.34	1.11E-03	3.34E-02	-0.97
	BAI2	0.30	8.67	3.34	1.13E-03	3.39E-02	-0.98
	RNF208	0.15	7.71	3.34	1.13E-03	3.39E-02	-0.99
	MMP24	0.21	7.91	3.33	1.14E-03	3.40E-02	-0.99
	LOC653596	0.16	7.73	3.33	1.15E-03	3.43E-02	-1.00
	VPS13C	0.28	8.17	3.33	1.16E-03	3.44E-02	-1.01
	CACNA1C	0.21	8.16	3.33	1.17E-03	3.44E-02	-1.01
	STH	0.24	7.90	3.33	1.17E-03	3.44E-02	-1.01
	HS.513971	0.28	9.17	3.33	1.17E-03	3.44E-02	-1.01
	LOC643423	0.17	7.72	3.32	1.18E-03	3.44E-02	-1.02
	GGTL3	0.22	8.19	3.32	1.18E-03	3.44E-02	-1.02
	HSF4	0.18	7.77	3.32	1.18E-03	3.44E-02	-1.02
	TTBK1	0.18	7.83	3.32	1.18E-03	3.44E-02	-1.02
	PPP2R5D	0.26	9.18	3.32	1.21E-03	3.50E-02	-1.05
	MACF1	0.27	7.90	3.31	1.25E-03	3.58E-02	-1.07
	RYR2	0.37	8.97	3.30	1.26E-03	3.60E-02	-1.08
	SAMD4B	0.36	8.49	3.30	1.27E-03	3.62E-02	-1.09
	ESRRA	0.23	8.20	3.30	1.28E-03	3.64E-02	-1.09
	C21ORF81	0.37	8.37	3.30	1.29E-03	3.64E-02	-1.10
	HS.560357	0.34	8.05	3.30	1.29E-03	3.64E-02	-1.10
	CSAD	0.14	7.70	3.30	1.29E-03	3.64E-02	-1.10
	HNT	0.35	11.31	3.30	1.30E-03	3.64E-02	-1.10
	PKD1	0.34	8.58	3.30	1.30E-03	3.64E-02	-1.10
	ITPK1	0.34	10.07	3.29	1.30E-03	3.64E-02	-1.11

	FAM73B	0.22	8.50	3.29	1.31E-03	3.64E-02	-1.12
	SLC4A3	0.29	8.74	3.29	1.32E-03	3.64E-02	-1.12
	OTUB1	0.41	9.23	3.29	1.32E-03	3.64E-02	-1.12
	HS.444999	0.29	7.95	3.29	1.32E-03	3.64E-02	-1.12
	PUS1	0.16	7.83	3.29	1.33E-03	3.67E-02	-1.13
	SHROOM4	0.68	10.56	3.29	1.34E-03	3.68E-02	-1.13
	GSDML	0.26	8.60	3.28	1.35E-03	3.68E-02	-1.14
	LOC649999	0.21	8.09	3.28	1.35E-03	3.70E-02	-1.14
	LOC92270	0.19	8.06	3.28	1.37E-03	3.71E-02	-1.15
	BTG1	0.30	9.75	3.28	1.37E-03	3.71E-02	-1.15
	HDAC8	0.19	7.86	3.28	1.37E-03	3.71E-02	-1.15
	THOC2	0.26	8.43	3.27	1.39E-03	3.72E-02	-1.17
	HSD11B1L	0.36	9.58	3.27	1.39E-03	3.72E-02	-1.17
	ATP1A3	0.30	7.96	3.27	1.42E-03	3.78E-02	-1.18
	ZNF362	0.24	9.30	3.26	1.43E-03	3.81E-02	-1.19
	TBC1D2B	0.23	8.38	3.26	1.44E-03	3.82E-02	-1.20
	SDHAP3	0.16	7.71	3.26	1.46E-03	3.83E-02	-1.21
	AES	0.35	11.54	3.26	1.48E-03	3.87E-02	-1.22
	KIAA0182	0.22	9.40	3.25	1.49E-03	3.89E-02	-1.22
	SLC25A29	0.32	8.45	3.25	1.49E-03	3.89E-02	-1.23
	QTRT1	0.25	8.13	3.25	1.51E-03	3.90E-02	-1.24
	WDR67	0.16	8.11	3.25	1.51E-03	3.90E-02	-1.24
	NBPF3	0.22	8.36	3.25	1.52E-03	3.92E-02	-1.24
	SNRPA	0.17	8.38	3.24	1.55E-03	3.98E-02	-1.26
	LOC440157	0.28	8.19	3.24	1.55E-03	3.98E-02	-1.26
	MGST1	0.38	7.93	3.24	1.57E-03	3.99E-02	-1.27
	C4ORF8	0.28	8.30	3.23	1.58E-03	3.99E-02	-1.28
	GFPT2	0.20	8.15	3.23	1.58E-03	3.99E-02	-1.28
	XRCC2	0.65	10.49	3.23	1.58E-03	3.99E-02	-1.28
	ZNF394	0.75	11.18	3.23	1.59E-03	3.99E-02	-1.28
	SCD5	0.19	7.98	3.23	1.59E-03	3.99E-02	-1.28
	MBD3	0.25	8.25	3.23	1.60E-03	3.99E-02	-1.29
	IFITM3	0.71	10.35	3.23	1.60E-03	3.99E-02	-1.29

	CCDC131	0.15	7.84	3.23	1.62E-03	4.00E-02	-1.30
	LOC732425	0.19	8.29	3.23	1.62E-03	4.00E-02	-1.30
	FLJ30092	0.20	7.80	3.23	1.62E-03	4.00E-02	-1.30
	SYT3	0.26	8.16	3.22	1.64E-03	4.01E-02	-1.31
	MT1X	0.88	10.46	3.22	1.64E-03	4.01E-02	-1.31
	FAM89B	0.32	9.44	3.22	1.66E-03	4.04E-02	-1.32
	EMP3	0.33	8.51	3.22	1.67E-03	4.06E-02	-1.33
	ZNF341	0.16	8.22	3.22	1.68E-03	4.06E-02	-1.33
	FIP1L1	0.24	8.77	3.21	1.68E-03	4.06E-02	-1.33
	KIAA1267	0.29	9.40	3.21	1.69E-03	4.06E-02	-1.34
	IFNAR2	0.22	7.99	3.21	1.69E-03	4.06E-02	-1.34
	ZNF280B	0.15	7.87	3.21	1.71E-03	4.09E-02	-1.35
	ST6GALNAC4	0.16	7.74	3.21	1.72E-03	4.11E-02	-1.35
	ZC3H12B	0.19	9.20	3.20	1.75E-03	4.15E-02	-1.37
	ZBTB40	0.28	8.29	3.20	1.77E-03	4.19E-02	-1.38
	USF2	0.39	8.74	3.20	1.79E-03	4.20E-02	-1.39
	MBTPS1	0.20	10.13	3.19	1.81E-03	4.25E-02	-1.40
	HYPK	0.68	11.97	3.19	1.83E-03	4.26E-02	-1.41
	DEAF1	0.35	9.98	3.18	1.85E-03	4.30E-02	-1.42
	HS.552826	0.16	7.77	3.18	1.90E-03	4.38E-02	-1.44
	TRIM11	0.15	7.93	3.18	1.90E-03	4.38E-02	-1.44
	LOC339879	0.29	7.88	3.17	1.91E-03	4.40E-02	-1.44
	TNFRSF1A	0.31	8.29	3.17	1.92E-03	4.40E-02	-1.45
	C1S	0.18	8.06	3.17	1.92E-03	4.40E-02	-1.45
	CPSF3L	0.18	8.65	3.17	1.93E-03	4.41E-02	-1.45
	AGPAT2	0.18	7.83	3.17	1.94E-03	4.41E-02	-1.46
	SPTBN4	0.45	9.24	3.17	1.96E-03	4.43E-02	-1.47
	MAGT1	0.51	12.00	3.16	1.97E-03	4.45E-02	-1.47
	CHPF	0.26	8.55	3.16	1.97E-03	4.45E-02	-1.47
	HS.184721	0.35	8.56	3.16	1.98E-03	4.46E-02	-1.48
	MED24	0.25	8.95	3.16	1.99E-03	4.46E-02	-1.48
	ARF1	0.25	8.64	3.16	1.99E-03	4.46E-02	-1.48
	PPFIA1	0.13	7.89	3.16	2.00E-03	4.47E-02	-1.48

	GRIPAP1	0.70	11.53	3.16	2.03E-03	4.52E-02	-1.50
	ASAHL	0.13	8.14	3.15	2.04E-03	4.52E-02	-1.50
	ADORA1	0.25	8.06	3.15	2.04E-03	4.52E-02	-1.50
	HS.542993	0.55	9.76	3.15	2.04E-03	4.52E-02	-1.50
	FLJ10769	0.21	10.09	3.15	2.05E-03	4.52E-02	-1.50
	PIGX	0.23	8.01	3.15	2.07E-03	4.55E-02	-1.52
	C14ORF133	0.16	8.11	3.15	2.08E-03	4.56E-02	-1.52
	LOC644284	0.17	7.73	3.15	2.08E-03	4.56E-02	-1.52
	CRTC1	0.24	7.98	3.14	2.10E-03	4.57E-02	-1.53
	STARD13	0.30	8.58	3.14	2.12E-03	4.59E-02	-1.54
	LOC644852	0.32	8.24	3.14	2.15E-03	4.60E-02	-1.55
	CLIC1	0.29	8.18	3.13	2.17E-03	4.61E-02	-1.56
	PDGFB	0.16	7.78	3.13	2.17E-03	4.61E-02	-1.56
	TIMP1	0.53	9.03	3.13	2.17E-03	4.61E-02	-1.56
	NDST1	0.26	8.30	3.13	2.19E-03	4.63E-02	-1.56
	MYO3B	0.36	8.27	3.13	2.20E-03	4.65E-02	-1.57
	FKRP	0.12	7.85	3.13	2.22E-03	4.66E-02	-1.57
	BLZF1	0.45	8.76	3.13	2.22E-03	4.66E-02	-1.57
	SCARA3	0.36	8.47	3.13	2.22E-03	4.67E-02	-1.58
	SPN	0.37	8.55	3.13	2.23E-03	4.67E-02	-1.58
	ZMYM3	0.23	8.38	3.13	2.23E-03	4.67E-02	-1.58
	MGC2752	0.15	7.86	3.12	2.24E-03	4.67E-02	-1.58
	WDR90	0.12	7.66	3.12	2.24E-03	4.68E-02	-1.58
	ARFRP1	0.14	7.73	3.12	2.25E-03	4.68E-02	-1.59
	MGC26718	0.20	7.97	3.12	2.25E-03	4.69E-02	-1.59
	CNNM1	0.27	9.26	3.12	2.27E-03	4.71E-02	-1.60
	RBM44	0.15	7.74	3.12	2.27E-03	4.71E-02	-1.60
	PDS5A	0.18	8.58	3.12	2.29E-03	4.71E-02	-1.60
	C19ORF29	0.18	7.92	3.12	2.30E-03	4.71E-02	-1.60
	NBPF11	0.28	9.61	3.12	2.30E-03	4.71E-02	-1.61
	ZYX	0.30	8.58	3.11	2.33E-03	4.77E-02	-1.62
	TMEM43	0.16	9.36	3.11	2.35E-03	4.78E-02	-1.62
	TAF1C	0.19	7.88	3.11	2.35E-03	4.78E-02	-1.63

	SELO	0.16	8.78	3.11	2.36E-03	4.80E-02	-1.63
	GSDML	0.22	8.11	3.11	2.37E-03	4.80E-02	-1.63
	C11ORF38	0.30	8.37	3.11	2.37E-03	4.80E-02	-1.63
	BST2	0.30	8.16	3.10	2.39E-03	4.81E-02	-1.64
	MTMR11	0.20	8.29	3.10	2.39E-03	4.81E-02	-1.64
	YY1	0.34	9.50	3.10	2.42E-03	4.85E-02	-1.65
	BRSK2	0.35	8.58	3.10	2.44E-03	4.89E-02	-1.66
	PNMA6A	0.32	8.89	3.10	2.45E-03	4.89E-02	-1.66
	RAF1	0.20	8.85	3.09	2.46E-03	4.89E-02	-1.66
	TMEM149	0.17	7.70	3.09	2.47E-03	4.90E-02	-1.67
	PLOD2	0.42	9.13	3.09	2.49E-03	4.93E-02	-1.68
	PDYN	0.18	7.85	3.09	2.49E-03	4.93E-02	-1.68
	ACTB	0.26	13.96	3.09	2.50E-03	4.94E-02	-1.68
	TFIP11	0.20	8.45	3.09	2.51E-03	4.94E-02	-1.68
	KIAA1715	0.29	9.41	3.09	2.51E-03	4.95E-02	-1.68
	G6PD	0.22	8.05	3.08	2.54E-03	4.99E-02	-1.69
	HOOK1	0.30	8.86	3.08	2.55E-03	5.00E-02	-1.70
	FHL1	0.27	10.00	3.08	2.56E-03	5.00E-02	-1.70
Down-regulation	*SST*	-1.38	10.21	-5.56	1.65E-07	9.35E-04	6.97
	CORT	-0.75	8.78	-5.47	2.51E-07	9.35E-04	6.59
	LOC644863	-0.49	12.73	-5.40	3.53E-07	9.35E-04	6.28
	TM2D1	-0.33	8.60	-5.21	7.89E-07	1.37E-03	5.55
	MRPL46	-0.41	9.60	-5.13	1.15E-06	1.37E-03	5.21
	CX3CR1	-0.94	8.75	-5.11	1.24E-06	1.37E-03	5.13
	PVALB	-0.92	10.02	-5.03	1.75E-06	1.58E-03	4.83
	TAC1	-0.69	8.59	-5.00	2.03E-06	1.68E-03	4.69
	KCNS3	-0.33	8.24	-4.95	2.44E-06	1.90E-03	4.52
	SLC32A1	-0.81	9.51	-4.93	2.73E-06	2.01E-03	4.42
	TAC1	-0.61	8.42	-4.90	3.01E-06	2.10E-03	4.33
	HINT1	-0.62	13.04	-4.88	3.36E-06	2.22E-03	4.23
	UQCRFS1	-0.50	12.33	-4.85	3.80E-06	2.39E-03	4.12
	LOC729466	-0.27	13.78	-4.81	4.44E-06	2.58E-03	3.98
	HS.552082	-0.56	8.97	-4.79	4.94E-06	2.58E-03	3.88

	CRHBP	-0.41	8.39	-4.77	5.25E-06	2.58E-03	3.83
	RAB33A	-0.51	9.13	-4.70	7.17E-06	3.26E-03	3.55
	DLX1	-0.55	9.13	-4.67	7.85E-06	3.26E-03	3.46
	ERP29	-0.36	11.57	-4.66	8.48E-06	3.26E-03	3.39
	CRH	-0.65	8.83	-4.65	8.62E-06	3.26E-03	3.38
	HACL1	-0.37	9.34	-4.59	1.12E-05	4.11E-03	3.14
	MRPS22	-0.25	10.29	-4.54	1.36E-05	4.72E-03	2.97
	C7ORF30	-0.40	10.44	-4.50	1.59E-05	5.27E-03	2.82
	HLA-DMB	-0.53	8.40	-4.48	1.75E-05	5.52E-03	2.74
	APEX1	-0.32	9.80	-4.44	1.99E-05	5.78E-03	2.62
	P2RY12	-0.71	8.38	-4.43	2.08E-05	5.78E-03	2.58
	FDFT1	-0.38	9.93	-4.43	2.11E-05	5.78E-03	2.57
	NDUFB6	-0.52	10.75	-4.43	2.12E-05	5.78E-03	2.57
	C10ORF32	-0.39	9.44	-4.42	2.15E-05	5.78E-03	2.55
	LOC341457	-0.51	14.27	-4.38	2.56E-05	6.36E-03	2.40
	PMM1	-0.30	10.66	-4.38	2.58E-05	6.36E-03	2.39
	APEX1	-0.35	10.54	-4.26	4.12E-05	8.07E-03	1.97
	CUTA	-0.41	11.73	-4.25	4.21E-05	8.07E-03	1.95
	PIN1	-0.36	12.91	-4.24	4.49E-05	8.48E-03	1.89
	SRP14	-0.36	12.73	-4.23	4.57E-05	8.51E-03	1.87
	MRPL36	-0.32	9.96	-4.23	4.67E-05	8.52E-03	1.85
	PSMB6	-0.47	11.74	-4.21	4.94E-05	8.52E-03	1.80
	GAD2	-0.57	9.62	-4.21	4.95E-05	8.52E-03	1.80
	DCTN3	-0.42	11.51	-4.21	5.08E-05	8.52E-03	1.78
	SH3KBP1	-0.35	10.37	-4.20	5.15E-05	8.52E-03	1.77
	HS.443490	-0.31	8.52	-4.20	5.16E-05	8.52E-03	1.76
	TMEM126A	-0.44	10.09	-4.20	5.28E-05	8.52E-03	1.74
	LARP6	-0.54	11.32	-4.18	5.57E-05	8.78E-03	1.70
	CCDC23	-0.35	10.11	-4.16	5.94E-05	9.25E-03	1.64
	COPS5	-0.38	11.24	-4.13	6.73E-05	1.01E-02	1.53
	CCDC115	-0.29	9.67	-4.12	7.16E-05	1.05E-02	1.47
	CSF1R	-0.92	9.64	-4.11	7.25E-05	1.05E-02	1.46
	TCEAL8	-0.37	9.64	-4.08	8.25E-05	1.16E-02	1.34

	GSTO1	-0.42	11.04	-4.07	8.34E-05	1.16E-02	1.33
	APOA1BP	-0.37	10.08	-4.05	9.04E-05	1.23E-02	1.26
	NPY	-0.94	10.59	-4.04	9.68E-05	1.27E-02	1.20
	MAD2L1BP	-0.33	8.82	-4.03	9.79E-05	1.27E-02	1.19
	COX5A	-0.39	12.49	-4.03	9.91E-05	1.27E-02	1.18
	THAP11	-0.24	9.98	-4.01	1.05E-04	1.29E-02	1.13
	STS-1	-0.51	9.44	-4.01	1.05E-04	1.29E-02	1.13
	PSMA6	-0.44	10.01	-4.00	1.10E-04	1.33E-02	1.09
	RELN	-0.43	8.27	-4.00	1.10E-04	1.33E-02	1.09
	ACAT1	-0.21	10.52	-4.00	1.10E-04	1.33E-02	1.08
	AMD1	-0.34	10.81	-3.98	1.20E-04	1.35E-02	1.01
	SNRPB2	-0.29	10.35	-3.98	1.21E-04	1.35E-02	1.00
	LOC653566	-0.30	10.50	-3.96	1.27E-04	1.36E-02	0.96
	GABARAPL2	-0.39	12.57	-3.96	1.29E-04	1.36E-02	0.94
	PTRH2	-0.28	9.37	-3.95	1.31E-04	1.37E-02	0.93
	CDC42	-0.47	8.50	-3.95	1.33E-04	1.37E-02	0.92
	TMEM85	-0.33	11.27	-3.94	1.39E-04	1.38E-02	0.87
	NDUFB2	-0.43	12.37	-3.93	1.44E-04	1.39E-02	0.85
	NFU1	-0.36	9.61	-3.91	1.55E-04	1.44E-02	0.78
	HS.412918	-0.35	8.17	-3.90	1.57E-04	1.44E-02	0.77
	MRPL21	-0.36	9.96	-3.89	1.63E-04	1.46E-02	0.74
	DCTN6	-0.31	10.19	-3.88	1.72E-04	1.50E-02	0.69
	TIMM23	-0.27	8.35	-3.88	1.73E-04	1.50E-02	0.68
	PTS	-0.39	9.35	-3.87	1.76E-04	1.51E-02	0.67
	THOC7	-0.36	11.24	-3.85	1.90E-04	1.61E-02	0.60
	MRPL20	-0.41	10.23	-3.85	1.91E-04	1.61E-02	0.59
	TBCA	-0.35	12.22	-3.81	2.25E-04	1.77E-02	0.45
	EXOSC1	-0.33	8.83	-3.81	2.25E-04	1.77E-02	0.45
	TGOLN2	-0.32	10.54	-3.80	2.29E-04	1.79E-02	0.43
	TMEM103	-0.25	9.65	-3.79	2.37E-04	1.81E-02	0.40
	FDFT1	-0.30	11.73	-3.79	2.41E-04	1.82E-02	0.38
	TM2D3	-0.22	8.06	-3.78	2.47E-04	1.82E-02	0.36
	C14ORF156	-0.46	12.27	-3.78	2.51E-04	1.82E-02	0.35

	NDUFS4	-0.38	11.00	-3.77	2.53E-04	1.82E-02	0.34
	PRDX2	-0.52	11.42	-3.77	2.58E-04	1.83E-02	0.33
	UTP14C	-0.25	9.06	-3.77	2.58E-04	1.83E-02	0.32
	COX7A2	-0.51	13.09	-3.76	2.64E-04	1.85E-02	0.30
	TRIM32	-0.30	8.56	-3.76	2.65E-04	1.85E-02	0.30
	CHMP5	-0.47	9.90	-3.75	2.71E-04	1.86E-02	0.28
	TMEM17	-0.25	8.07	-3.74	2.80E-04	1.89E-02	0.25
	C3ORF68	-0.23	8.22	-3.74	2.84E-04	1.90E-02	0.24
	MRPS18C	-0.54	10.60	-3.73	2.92E-04	1.91E-02	0.22
	RTN4	-0.93	10.55	-3.73	2.97E-04	1.92E-02	0.20
	SLCO2B1	-0.52	9.68	-3.72	3.02E-04	1.94E-02	0.18
	GAD1	-0.74	11.94	-3.72	3.05E-04	1.94E-02	0.17
	C21ORF51	-0.25	8.88	-3.72	3.07E-04	1.94E-02	0.17
	C2ORF25	-0.38	9.83	-3.72	3.08E-04	1.94E-02	0.17
	MAF	-0.27	8.18	-3.71	3.11E-04	1.94E-02	0.16
	KIAA0859	-0.30	8.79	-3.71	3.13E-04	1.94E-02	0.15
	TMEM177	-0.24	8.72	-3.71	3.19E-04	1.94E-02	0.14
	ASH2L	-0.27	10.17	-3.71	3.20E-04	1.94E-02	0.13
	SCO2	-0.36	8.99	-3.71	3.21E-04	1.94E-02	0.13
	NXPH1	-0.43	9.18	-3.69	3.41E-04	1.97E-02	0.08
	PTP4A1	-0.35	8.45	-3.68	3.48E-04	2.00E-02	0.06
	UCRC	-0.37	9.53	-3.68	3.52E-04	2.00E-02	0.05
	RPAIN	-0.30	9.09	-3.68	3.53E-04	2.00E-02	0.04
	FEN1	-0.24	8.20	-3.68	3.55E-04	2.00E-02	0.04
	LOC643668	-0.55	10.36	-3.67	3.69E-04	2.01E-02	0.01
	ANKS1A	-0.22	9.79	-3.66	3.71E-04	2.01E-02	0.00
	PPA1	-0.36	12.35	-3.66	3.79E-04	2.03E-02	-0.02
	TRPT1	-0.37	10.08	-3.65	3.96E-04	2.08E-02	-0.06
	IHPK2	-0.25	8.26	-3.65	3.97E-04	2.08E-02	-0.06
	FBXW11	-0.21	11.09	-3.64	4.03E-04	2.09E-02	-0.07
	CCNH	-0.22	8.53	-3.63	4.19E-04	2.14E-02	-0.11
	PSMA5	-0.32	10.21	-3.63	4.21E-04	2.14E-02	-0.11
	LOC647340	-0.31	12.01	-3.62	4.28E-04	2.16E-02	-0.13

	JTV1	-0.23	9.54	-3.62	4.34E-04	2.18E-02	-0.14
	RPA3	-0.36	9.49	-3.62	4.40E-04	2.18E-02	-0.15
	C3ORF10	-0.36	9.75	-3.62	4.40E-04	2.18E-02	-0.15
	KIAA1737	-0.26	10.63	-3.61	4.54E-04	2.23E-02	-0.18
	NDUFA3	-0.43	12.09	-3.61	4.55E-04	2.23E-02	-0.18
	HS.537004	-0.46	9.89	-3.60	4.61E-04	2.23E-02	-0.19
	C15ORF24	-0.26	11.20	-3.60	4.61E-04	2.23E-02	-0.19
	PSMD7	-0.41	10.76	-3.60	4.65E-04	2.24E-02	-0.20
	ARMC10	-0.40	10.25	-3.59	4.81E-04	2.30E-02	-0.23
	FLJ35801	-0.21	7.93	-3.58	4.93E-04	2.32E-02	-0.25
	SLC25A26	-0.26	7.98	-3.58	4.99E-04	2.34E-02	-0.26
	TFCP2	-0.21	9.04	-3.58	5.02E-04	2.35E-02	-0.27
	COPS7A	-0.24	11.59	-3.58	5.07E-04	2.35E-02	-0.28
	MGC27121	-0.30	8.27	-3.56	5.27E-04	2.40E-02	-0.31
	MRPS9	-0.27	9.76	-3.56	5.34E-04	2.40E-02	-0.32
	ZMAT2	-0.38	10.46	-3.56	5.36E-04	2.40E-02	-0.32
	KHDRBS1	-0.34	10.78	-3.55	5.44E-04	2.42E-02	-0.34
	RAD51C	-0.27	9.06	-3.55	5.47E-04	2.43E-02	-0.34
	MRPL22	-0.27	8.82	-3.55	5.49E-04	2.43E-02	-0.35
	COX17	-0.44	11.45	-3.55	5.51E-04	2.43E-02	-0.35
	MORN2	-0.31	8.94	-3.55	5.53E-04	2.43E-02	-0.35
	BCL11A	-0.31	8.30	-3.55	5.56E-04	2.43E-02	-0.36
	PSMC6	-0.43	10.58	-3.55	5.58E-04	2.43E-02	-0.36
	STARD7	-0.26	11.90	-3.55	5.58E-04	2.43E-02	-0.36
	SIP1	-0.29	8.19	-3.54	5.67E-04	2.44E-02	-0.37
	TCTEX1D2	-0.40	10.60	-3.54	5.73E-04	2.46E-02	-0.38
	CRSP9	-0.24	8.28	-3.53	5.87E-04	2.49E-02	-0.41
	CHCHD9	-0.32	10.99	-3.53	5.91E-04	2.49E-02	-0.41
	RPL26L1	-0.28	8.54	-3.53	6.00E-04	2.52E-02	-0.43
	PRG-3	-0.48	9.46	-3.52	6.21E-04	2.54E-02	-0.46
	CCT2	-0.39	10.52	-3.51	6.31E-04	2.56E-02	-0.47
	CRYM	-0.58	9.02	-3.51	6.38E-04	2.56E-02	-0.48
	LCMT1	-0.34	9.49	-3.51	6.39E-04	2.56E-02	-0.48

	COMMD3	-0.24	10.78	-3.50	6.51E-04	2.57E-02	-0.50
	SNURF	-0.65	12.54	-3.50	6.65E-04	2.61E-02	-0.52
	MRPS11	-0.28	9.43	-3.49	6.81E-04	2.64E-02	-0.54
	SPCS2	-0.25	10.93	-3.48	7.02E-04	2.71E-02	-0.56
	HIGD1A	-0.42	11.38	-3.48	7.06E-04	2.71E-02	-0.57
	SLC25A5	-0.25	12.59	-3.48	7.09E-04	2.71E-02	-0.57
	PIPSL	-0.33	9.31	-3.48	7.12E-04	2.71E-02	-0.58
	TCEAL7	-0.51	10.65	-3.47	7.29E-04	2.76E-02	-0.60
	LOC729101	-0.20	7.95	-3.47	7.33E-04	2.76E-02	-0.60
	UROD	-0.19	10.76	-3.46	7.45E-04	2.79E-02	-0.62
	PREI3	-0.44	10.25	-3.46	7.48E-04	2.79E-02	-0.62
	KCNK1	-0.59	9.99	-3.46	7.51E-04	2.79E-02	-0.62
	SYT11	-0.36	14.36	-3.46	7.55E-04	2.79E-02	-0.63
	SLCO2A1	-0.25	8.16	-3.45	7.68E-04	2.83E-02	-0.64
	MRPL40	-0.18	9.02	-3.45	7.80E-04	2.86E-02	-0.66
	ENTPD3	-0.38	8.61	-3.44	8.07E-04	2.93E-02	-0.69
	PGM5	-0.27	8.13	-3.43	8.18E-04	2.95E-02	-0.70
	LOC653479	-0.32	8.72	-3.43	8.22E-04	2.95E-02	-0.70
	LOC651143	-0.24	8.19	-3.43	8.24E-04	2.95E-02	-0.71
	PIH1D1	-0.19	8.70	-3.43	8.31E-04	2.96E-02	-0.71
	PSMB3	-0.28	11.08	-3.43	8.33E-04	2.96E-02	-0.72
	PPIE	-0.18	8.58	-3.43	8.40E-04	2.96E-02	-0.72
	ETV5	-0.46	10.87	-3.43	8.41E-04	2.96E-02	-0.72
	NDUFA12	-0.39	11.29	-3.43	8.42E-04	2.96E-02	-0.72
	TXN	-0.49	12.18	-3.42	8.45E-04	2.96E-02	-0.73
	UBE2E3	-0.33	8.94	-3.42	8.49E-04	2.96E-02	-0.73
	COPS4	-0.44	9.97	-3.42	8.52E-04	2.96E-02	-0.74
	HS.576633	-0.31	8.46	-3.42	8.62E-04	2.97E-02	-0.75
	ASMTL	-0.27	8.94	-3.42	8.66E-04	2.97E-02	-0.75
	C6ORF66	-0.38	8.64	-3.41	8.75E-04	2.99E-02	-0.76
	PRDX2	-0.36	10.90	-3.41	8.80E-04	3.00E-02	-0.76
	BOLA3	-0.30	10.92	-3.41	8.99E-04	3.04E-02	-0.78
	PDCD2	-0.25	8.93	-3.40	9.06E-04	3.05E-02	-0.79

	PFN1	-0.29	11.79	-3.40	9.12E-04	3.05E-02	-0.80
	HNRPK	-0.40	11.12	-3.40	9.26E-04	3.05E-02	-0.81
	EEF1B2	-0.37	9.41	-3.40	9.29E-04	3.05E-02	-0.81
	LOC653226	-0.49	10.98	-3.40	9.31E-04	3.05E-02	-0.81
	DCTN2	-0.29	12.20	-3.40	9.32E-04	3.05E-02	-0.81
	BEX5	-0.61	11.07	-3.39	9.38E-04	3.06E-02	-0.82
	CLASP2	-0.55	10.19	-3.39	9.47E-04	3.07E-02	-0.83
	NHP2L1	-0.48	10.46	-3.39	9.51E-04	3.08E-02	-0.83
	DSCR3	-0.23	8.91	-3.39	9.56E-04	3.09E-02	-0.84
	PCP4	-0.59	11.18	-3.39	9.61E-04	3.09E-02	-0.84
	LHX6	-0.40	9.66	-3.38	9.77E-04	3.12E-02	-0.86
	WDR61	-0.25	10.18	-3.38	9.91E-04	3.13E-02	-0.87
	NDUFB6	-0.36	9.88	-3.38	9.93E-04	3.13E-02	-0.87
	PDHB	-0.27	11.85	-3.37	1.00E-03	3.13E-02	-0.88
	TCEAL1	-0.36	9.32	-3.37	1.01E-03	3.15E-02	-0.89
	TUBG1	-0.31	9.91	-3.37	1.02E-03	3.15E-02	-0.89
	KIAA0859	-0.24	9.53	-3.36	1.05E-03	3.23E-02	-0.92
	CABYR	-0.22	8.48	-3.36	1.06E-03	3.23E-02	-0.93
	UQCRH	-0.45	12.98	-3.35	1.09E-03	3.31E-02	-0.95
	PDCD6	-0.24	11.35	-3.35	1.09E-03	3.31E-02	-0.95
	PSME4	-0.29	8.74	-3.34	1.11E-03	3.35E-02	-0.97
	ERP29	-0.32	8.86	-3.34	1.13E-03	3.39E-02	-0.99
	FUS	-0.16	8.46	-3.33	1.17E-03	3.44E-02	-1.01
	GPATCH4	-0.32	9.26	-3.32	1.18E-03	3.44E-02	-1.02
	VIP	-0.56	8.86	-3.32	1.19E-03	3.46E-02	-1.03
	TSNAX	-0.47	9.40	-3.32	1.20E-03	3.47E-02	-1.04
	RPUSD3	-0.24	9.00	-3.31	1.23E-03	3.54E-02	-1.06
	PSMA1	-0.33	10.93	-3.31	1.23E-03	3.54E-02	-1.06
	STRN3	-0.33	9.44	-3.30	1.26E-03	3.60E-02	-1.08
	C20ORF39	-0.35	9.85	-3.30	1.29E-03	3.64E-02	-1.10
	LOC645058	-0.35	10.70	-3.29	1.31E-03	3.64E-02	-1.11
	C20ORF116	-0.29	10.39	-3.29	1.31E-03	3.64E-02	-1.11
	ACAT2	-0.36	10.03	-3.29	1.32E-03	3.64E-02	-1.12

	PENK	-0.38	8.13	-3.29	1.34E-03	3.68E-02	-1.13
	GALT	-0.23	9.15	-3.28	1.35E-03	3.68E-02	-1.14
	RNF103	-0.24	10.55	-3.28	1.36E-03	3.70E-02	-1.15
	MRPL9	-0.17	8.23	-3.28	1.37E-03	3.71E-02	-1.16
	ATP5C1	-0.41	10.43	-3.28	1.38E-03	3.72E-02	-1.16
	ACP1	-0.47	10.83	-3.28	1.38E-03	3.72E-02	-1.16
	SQLE	-0.28	8.55	-3.27	1.39E-03	3.72E-02	-1.17
	RGS10	-0.34	8.12	-3.27	1.43E-03	3.81E-02	-1.19
	HIAT1	-0.34	8.91	-3.26	1.43E-03	3.81E-02	-1.19
	LANCL1	-0.42	12.06	-3.26	1.45E-03	3.82E-02	-1.20
	HNMT	-0.18	7.97	-3.26	1.46E-03	3.83E-02	-1.21
	PSMC4	-0.39	8.81	-3.26	1.47E-03	3.87E-02	-1.22
	DCUN1D1	-0.27	8.47	-3.25	1.49E-03	3.89E-02	-1.23
	FEZ1	-0.39	9.85	-3.25	1.50E-03	3.89E-02	-1.23
	LOC644096	-0.21	9.39	-3.25	1.50E-03	3.90E-02	-1.24
	MRPS33	-0.36	9.83	-3.25	1.51E-03	3.90E-02	-1.24
	TF	-0.85	12.76	-3.24	1.55E-03	3.97E-02	-1.26
	PDCL3	-0.21	9.00	-3.24	1.56E-03	3.98E-02	-1.27
	SLTM	-0.19	9.78	-3.24	1.56E-03	3.99E-02	-1.27
	TMEM14A	-0.49	10.89	-3.24	1.57E-03	3.99E-02	-1.27
	PWP1	-0.23	9.72	-3.23	1.60E-03	3.99E-02	-1.29
	NDUFA8	-0.28	12.22	-3.23	1.60E-03	3.99E-02	-1.29
	CYP26A1	-0.25	8.00	-3.23	1.60E-03	3.99E-02	-1.29
	UBE2E3	-0.33	10.45	-3.23	1.61E-03	3.99E-02	-1.29
	PSMC4	-0.39	8.76	-3.23	1.61E-03	3.99E-02	-1.29
	NGDN	-0.21	8.60	-3.23	1.62E-03	4.00E-02	-1.30
	PRPS1	-0.30	9.84	-3.22	1.63E-03	4.01E-02	-1.31
	HBXIP	-0.34	11.58	-3.22	1.64E-03	4.01E-02	-1.31
	MTERFD1	-0.23	9.01	-3.22	1.64E-03	4.01E-02	-1.31
	TMTC4	-0.35	8.31	-3.22	1.66E-03	4.04E-02	-1.32
	LOC643668	-0.36	8.54	-3.21	1.69E-03	4.06E-02	-1.34
	DDX1	-0.33	11.84	-3.21	1.69E-03	4.06E-02	-1.34
	TAC3	-0.37	8.62	-3.21	1.72E-03	4.11E-02	-1.35

	CTCF	-0.24	8.99	-3.21	1.73E-03	4.12E-02	-1.36
	SUMO2	-0.38	10.96	-3.20	1.74E-03	4.13E-02	-1.36
	HSPA8	-0.50	13.03	-3.20	1.74E-03	4.13E-02	-1.36
	NSF	-0.76	10.17	-3.20	1.76E-03	4.16E-02	-1.37
	TMEM126B	-0.31	10.20	-3.20	1.76E-03	4.17E-02	-1.37
	LYPLAL1	-0.20	8.37	-3.20	1.79E-03	4.20E-02	-1.39
	THYN1	-0.29	9.79	-3.19	1.82E-03	4.25E-02	-1.40
	MMACHC	-0.18	8.07	-3.19	1.82E-03	4.25E-02	-1.40
	TMEM16D	-0.24	8.54	-3.19	1.82E-03	4.25E-02	-1.40
	OAT	-0.35	11.09	-3.19	1.83E-03	4.26E-02	-1.41
	PTTG1	-0.23	8.02	-3.18	1.89E-03	4.37E-02	-1.43
	HS.538100	-0.31	8.71	-3.18	1.89E-03	4.38E-02	-1.44
	EXOC1	-0.24	9.12	-3.17	1.92E-03	4.40E-02	-1.45
	RPS24	-0.34	9.26	-3.17	1.93E-03	4.41E-02	-1.45
	INSIG1	-0.34	10.31	-3.17	1.94E-03	4.42E-02	-1.46
	RPL6	-0.33	8.56	-3.17	1.96E-03	4.43E-02	-1.47
	KIT	-0.34	9.08	-3.17	1.96E-03	4.43E-02	-1.47
	HS.390250	-0.58	10.86	-3.16	1.99E-03	4.47E-02	-1.48
	C2ORF47	-0.22	9.13	-3.16	2.00E-03	4.47E-02	-1.49
	VIP	-0.55	10.29	-3.15	2.04E-03	4.52E-02	-1.50
	PRMT6	-0.21	8.73	-3.15	2.04E-03	4.52E-02	-1.50
	C2ORF25	-0.36	10.14	-3.15	2.06E-03	4.53E-02	-1.51
	VPS24	-0.34	8.75	-3.15	2.06E-03	4.53E-02	-1.51
	NDUFAF2	-0.25	9.77	-3.15	2.09E-03	4.56E-02	-1.52
	NINJ2	-0.70	9.28	-3.14	2.10E-03	4.57E-02	-1.53
	C6ORF159	-0.26	8.76	-3.14	2.11E-03	4.58E-02	-1.53
	ARMCX3	-0.40	9.15	-3.14	2.11E-03	4.58E-02	-1.53
	CHMP5	-0.34	9.92	-3.14	2.12E-03	4.59E-02	-1.53
	TMEM157	-0.37	9.95	-3.14	2.12E-03	4.59E-02	-1.54
	SCHIP1	-0.32	11.11	-3.14	2.13E-03	4.59E-02	-1.54
	UQCRQ	-0.32	12.79	-3.14	2.14E-03	4.60E-02	-1.54
	ATP5H	-0.37	12.60	-3.14	2.14E-03	4.60E-02	-1.54
	COPA	-0.17	11.27	-3.14	2.15E-03	4.60E-02	-1.55

	RFX5	-0.23	9.47	-3.14	2.15E-03	4.60E-02	-1.55
	CCDC25	-0.31	11.08	-3.13	2.17E-03	4.61E-02	-1.56
	HS.548045	-0.22	8.33	-3.13	2.17E-03	4.61E-02	-1.56
	LOC389895	-0.23	8.57	-3.13	2.18E-03	4.62E-02	-1.56
	TMEM14C	-0.22	10.20	-3.12	2.28E-03	4.71E-02	-1.60
	ARHGDIB	-0.40	10.00	-3.12	2.28E-03	4.71E-02	-1.60
	NDUFB5	-0.26	12.05	-3.12	2.28E-03	4.71E-02	-1.60
	RNF5P1	-0.16	8.39	-3.12	2.29E-03	4.71E-02	-1.60
	TM6SF1	-0.37	9.28	-3.11	2.31E-03	4.73E-02	-1.61
	GPR37	-0.79	10.86	-3.11	2.32E-03	4.74E-02	-1.61
	VAMP7	-0.27	9.88	-3.11	2.34E-03	4.77E-02	-1.62
	KIAA1279	-0.32	10.19	-3.10	2.38E-03	4.81E-02	-1.64
	CCNDBP1	-0.34	11.36	-3.10	2.39E-03	4.81E-02	-1.64
	APITD1	-0.19	8.02	-3.10	2.42E-03	4.85E-02	-1.65
	AUH	-0.35	9.52	-3.10	2.44E-03	4.89E-02	-1.66
	DOCK10	-0.49	9.78	-3.09	2.47E-03	4.90E-02	-1.67
	TRAPPC4	-0.28	10.11	-3.09	2.48E-03	4.92E-02	-1.67
	CDC123	-0.20	9.59	-3.09	2.53E-03	4.98E-02	-1.69
	SLC10A4	-0.15	7.72	-3.08	2.55E-03	5.00E-02	-1.70
	RNF5P1	-0.23	10.14	-3.08	2.56E-03	5.00E-02	-1.70

Table S3. Functional enrichment of differentially expressed genes.

Expression	Term	Fold Enrichment	*p*-Value	Benjamini *Q*-Value
Up-regulation	hsa04010: MAPK signaling pathway	3.56	1.36E-05	1.48E-03
	hsa04510: focal adhesion	3.89	4.36E-05	2.38E-03
	GO:0007243: protein kinase cascade	2.80	2.47E-04	3.61E-01
	GO:0008134: transcription factor binding	2.41	3.11E-04	1.35E-01
	GO:0005794: Golgi apparatus	2.08	3.65E-04	1.17E-01
	GO:0031328: positive regulation of cellular biosynthetic process	2.02	1.81E-03	8.06E-01
	GO:0044431: Golgi apparatus part	2.87	1.94E-03	2.83E-01
	GO:0009891: positive regulation of biosynthetic process	1.99	2.17E-03	7.31E-01
	GO:0010557: positive regulation of macromolecule biosynthetic process	2.02	2.22E-03	6.35E-01
	GO:0006357: regulation of transcription from RNA polymerase II promoter	1.90	3.80E-03	7.49E-01
	GO:0012505: endomembrane system	1.91	4.28E-03	3.87E-01
	GO:0044451: nucleoplasm part	2.10	5.19E-03	3.59E-01
	GO:0016192: vesicle-mediated transport	2.00	5.40E-03	8.05E-01

	GO:0007218: neuropeptide signaling pathway	4.33	5.48E-03	7.59E-01
	GO:0006468: protein amino acid phosphorylation	1.90	5.85E-03	7.35E-01
	GO:0030863: cortical cytoskeleton	6.62	6.63E-03	3.66E-01
	GO:0005938: cell cortex	3.56	7.25E-03	3.40E-01
	hsa04912: GnRH signaling pathway	3.99	7.43E-03	2.37E-01
	GO:0051173: positive regulation of nitrogen compound metabolic process	1.88	8.47E-03	8.20E-01
	GO:0044448: cell cortex part	4.69	8.91E-03	3.54E-01
	GO:0007010: cytoskeleton organization	2.11	8.96E-03	8.04E-01
	GO:0044420: extracellular matrix part	3.88	9.24E-03	3.27E-01
	GO:0004674: protein serine/threonine kinase activity	2.09	9.71E-03	8.97E-01
	GO:0008361: regulation of cell size	2.79	9.71E-03	8.00E-01
	GO:0019717: synaptosome	4.58	9.82E-03	3.13E-01
Down-regulation	GO:0005739: mitochondrion	3.21	8.63E-14	2.49E-11
	GO:0044429: mitochondrial part	3.56	2.20E-09	3.18E-07
	GO:0030529: ribonucleoprotein complex	3.72	6.76E-09	6.51E-07
	hsa05012: Parkinson's disease	6.77	2.29E-08	2.22E-06
	hsa00190: oxidative phosphorylation	6.67	2.80E-08	1.36E-06
	hsa03050: proteasome	12.29	6.62E-08	2.14E-06
	GO:0070469: respiratory chain	10.03	1.22E-07	8.82E-06
	GO:0000502: proteasome complex	11.21	2.18E-07	1.26E-05
	GO:0016655: oxidoreductase activity acting on NADH or NADPH, quinone, or similar compound as acceptor	13.70	2.39E-07	1.06E-04
	GO:0000313: organellar ribosome	12.82	3.97E-07	1.91E-05
	GO:0005761: mitochondrial ribosome	12.82	3.97E-07	1.91E-05
	GO:0051443: positive regulation of ubiquitin-protein ligase activity	10.39	4.37E-07	5.27E-04
	GO:0005743: mitochondrial inner membrane	4.24	5.09E-07	2.10E-05
	GO:0005840: ribosome	5.09	5.61E-07	2.02E-05
	GO:0051351: positive regulation of ligase activity	9.96	6.29E-07	3.79E-04
	GO:0051438: regulation of ubiquitin-protein ligase activity	9.32	1.11E-06	4.48E-04
	GO:0050136: NADH dehydrogenase (quinone) activity	13.88	1.42E-06	3.13E-04
	GO:0008137: NADH dehydrogenase (ubiquinone) activity	13.88	1.42E-06	3.13E-04
	GO:0003954: NADH dehydrogenase activity	13.88	1.42E-06	3.13E-04
	GO:0019866: organelle inner membrane	3.95	1.46E-06	4.68E-05
	GO:0051340: regulation of ligase activity	8.98	1.54E-06	4.64E-04
	hsa05016: Huntington's disease	4.82	1.65E-06	3.99E-05
	GO:0044455: mitochondrial membrane part	6.56	2.02E-06	5.85E-05
	GO:0031398: positive regulation of protein ubiquitination	8.66	2.10E-06	5.06E-04
	GO:0003735: structural constituent of ribosome	5.77	2.49E-06	3.67E-04
	GO:0051436: negative regulation of ubiquitin-protein ligase activity during mitotic cell cycle	10.07	2.73E-06	5.48E-04
	GO:0031145: anaphase-promoting complex-dependent proteasomal ubiquitin-dependent protein catabolic process	10.07	2.73E-06	5.48E-04
	GO:0051444: negative regulation of ubiquitin-protein ligase activity	9.77	3.44E-06	5.93E-04
	GO:0051352: negative regulation of ligase activity	9.77	3.44E-06	5.93E-04

GO:0022900: electron transport chain	7.02	3.57E-06	5.38E-04
GO:0005746: mitochondrial respiratory chain	9.61	3.82E-06	1.00E-04
GO:0051437: positive regulation of ubiquitin-protein ligase activity during mitotic cell cycle	9.63	3.86E-06	5.17E-04
GO:0051439: regulation of ubiquitin-protein ligase activity during mitotic cell cycle	9.22	5.36E-06	6.46E-04
GO:0031397: negative regulation of protein ubiquitination	8.85	7.33E-06	8.04E-04
GO:0031396: regulation of protein ubiquitination	7.27	8.98E-06	9.03E-04
GO:0005740: mitochondrial envelope	3.26	1.14E-05	2.74E-04
GO:0016651: oxidoreductase activity acting on NADH or NADPH	8.09	1.42E-05	1.57E-03
GO:0033279: ribosomal subunit	5.87	1.72E-05	3.82E-04
hsa05010: Alzheimer's disease	4.61	1.73E-05	3.35E-04
GO:0031966: mitochondrial membrane	3.30	1.77E-05	3.66E-04
GO:0031980: mitochondrial lumen	4.22	2.75E-05	5.30E-04
GO:0005759: mitochondrial matrix	4.22	2.75E-05	5.30E-04
GO:0006119: oxidative phosphorylation	6.68	5.79E-05	5.36E-03
GO:0010498: proteasomal protein catabolic process	6.42	7.71E-05	6.62E-03
GO:0043161: proteasomal ubiquitin-dependent protein catabolic process	6.42	7.71E-05	6.62E-03
GO:0006091: generation of precursor metabolites and energy	3.49	1.03E-04	8.23E-03
GO:0042775: mitochondrial ATP synthesis coupled electron transport	9.09	1.11E-04	8.33E-03
GO:0042773: ATP synthesis coupled electron transport	9.09	1.11E-04	8.33E-03
GO:0005763: mitochondrial small ribosomal subunit	18.99	1.13E-04	2.04E-03
GO:0000314: organellar small ribosomal subunit	18.99	1.13E-04	2.04E-03
GO:0070003: threonine-type peptidase activity	18.65	1.25E-04	1.10E-02
GO:0004298: threonine-type endopeptidase activity	18.65	1.25E-04	1.10E-02
GO:0005839: proteasome core complex	17.09	1.75E-04	2.98E-03
GO:0006412: translation	3.30	1.86E-04	1.31E-02
GO:0005184: neuropeptide hormone activity	16.22	2.21E-04	1.61E-02
GO:0031400: negative regulation of protein modification process	5.50	2.26E-04	1.51E-02
GO:0022904: respiratory electron transport chain	7.95	2.34E-04	1.48E-02
GO:0006120: mitochondrial electron transport of NADH to ubiquinone	10.39	2.56E-04	1.53E-02
GO:0030964: NADH dehydrogenase complex	9.76	3.40E-04	5.44E-03
GO:0005747: mitochondrial respiratory chain complex I	9.76	3.40E-04	5.44E-03
GO:0045271: respiratory chain complex I	9.76	3.40E-04	5.44E-03
GO:0045333: cellular respiration	6.00	3.64E-04	2.07E-02
GO:0031090: organelle membrane	1.93	5.42E-04	8.21E-03
GO:0000278: mitotic cell cycle	2.95	5.69E-04	3.07E-02
GO:0031967: organelle envelope	2.32	7.03E-04	1.01E-02
GO:0031975: envelope	2.31	7.32E-04	1.00E-02
GO:0032269: negative regulation of cellular protein metabolic process	4.04	8.28E-04	4.25E-02
GO:0007049: cell cycle	2.16	9.27E-04	4.56E-02

Table S4. Functional enrichment of differentially expressed genes (MITHrIL)

Expression	Term	*P*-Value	Impact Factor	Perturbation Accumulation	Raw Accumulation	Total Perturbation	Probability
Up-regulation	hsa04510:Focal adhesion	1.40E-06	24.23	1.65	5.04	2.13	22.10
	hsa05215:Prostate cancer	3.22E-06	6.75	1.54	2.20	3.06	3.70
	hsa04910:Insulin signaling pathway	1.45E-05	13.33	5.25	6.08	3.56	9.77
	hsa04010:MAPK signaling pathway	2.11E-05	27.57	0.26	1.71	1.65	25.92
	hsa04650:Natural killer cell mediated cytotoxicity	3.53E-05	10.67	5.20	6.52	4.88	5.79
	hsa05200:Pathways in cancer	5.53E-05	13.90	5.86	10.90	4.84	9.06
	hsa05221:Acute myeloid leukemia	1.72E-04	9.40	1.32	1.74	2.13	7.27
	hsa05214:Glioma	3.63E-04	11.82	2.48	5.60	5.05	6.78
	hsa05034:Alcoholism	4.53E-04	14.80	1.85	5.05	2.67	12.14
	hsa04540:Gap junction	4.66E-04	10.67	1.56	2.12	2.89	7.78
	hsa04115:p53 signaling pathway	5.48E-04	6.64	-0.96	-0.96	2.24	4.41
	hsa04724:Glutamatergic synapse	7.58E-04	12.76	-1.69	-1.79	1.93	10.83
	hsa04810:Regulation of actin cytoskeleton	1.01E-03	8.10	-1.41	-0.13	0.98	7.12
	hsa04012:ErbB signaling pathway	1.02E-03	11.72	2.25	4.50	3.84	7.89
	hsa04713:Circadian entrainment	1.10E-03	12.25	1.33	2.72	2.55	9.70
	hsa04660:T cell receptor signaling pathway	1.31E-03	5.74	1.47	1.67	2.54	3.20
	hsa04725:Cholinergic synapse	1.42E-03	8.93	-1.77	0.33	2.19	6.74
	hsa04912:GnRH signaling pathway	1.96E-03	14.57	0.71	2.68	2.15	12.42
	hsa04727:GABAergic synapse	2.58E-03	11.87	-0.76	-1.30	1.75	10.13
	hsa05146:Amoebiasis	3.14E-03	6.88	-0.98	-0.68	1.97	4.91
	hsa04723:Retrograde endocannabinoid signaling	3.52E-03	11.02	-0.60	-0.10	1.66	9.36
	hsa04512:ECM-receptor interaction	4.24E-03	8.23	0.71	1.63	2.49	5.75
	hsa05223:Non-small cell lung cancer	4.29E-03	7.26	1.78	2.26	4.36	2.90
	hsa05152:Tuberculosis	4.69E-03	7.64	2.58	3.78	4.36	3.28
	hsa04151:PI3K-Akt signaling pathway	5.12E-03	18.96	0.42	9.07	3.43	15.53
	hsa04066:HIF-1 signaling pathway	6.47E-03	8.45	-1.28	0.53	1.46	6.98
	hsa05218:Melanoma	6.75E-03	8.30	1.33	2.64	4.01	4.29
	hsa05211:Renal cell carcinoma	7.32E-03	5.95	0.60	0.84	1.46	4.49
	hsa05133:Pertussis	8.77E-03	5.80	-0.45	-0.13	1.65	4.14

	hsa05145:Toxoplasmosis	9.08E-03	8.29	1.50	1.88	3.65	4.64
	hsa04380:Osteoclast differentiation	1.02E-02	6.12	0.84	1.33	1.89	4.23
	hsa05032:Morphine addiction	1.03E-02	9.16	-0.54	0.44	1.53	7.63
	hsa05213:Endometrial cancer	1.06E-02	8.21	1.51	2.04	3.06	5.14
	hsa04664:Fc epsilon RI signaling pathway	1.06E-02	7.24	1.44	2.04	2.91	4.33
	hsa05166:HTLV-I infection	1.15E-02	5.63	1.58	2.34	2.39	3.25
	hsa05014:Amyotrophic lateral sclerosis (ALS)	1.17E-02	7.29	1.10	1.24	2.25	5.04
	hsa04911:Insulin secretion	1.28E-02	9.61	-0.66	1.23	1.72	7.89
	hsa04060:Cytokine-cytokine receptor interaction	1.39E-02	4.59	-0.93	0.16	1.35	3.23
	hsa04726:Serotonergic synapse	1.50E-02	9.20	0.62	1.46	2.50	6.70
	hsa05220:Chronic myeloid leukemia	1.57E-02	6.47	0.92	1.36	2.26	4.22
	hsa05205:Proteoglycans in cancer	1.93E-02	9.60	0.91	3.92	2.82	6.78
	hsa04390:Hippo signaling pathway	2.33E-02	6.36	-2.17	-0.97	3.99	2.36
	hsa04728:Dopaminergic synapse	2.69E-02	12.89	-0.13	0.85	2.74	10.15
	hsa04370:VEGF signaling pathway	2.84E-02	9.73	0.53	2.09	3.07	6.66
	hsa05010:Alzheimer's disease	3.48E-02	3.95	0.93	0.93	1.81	2.14
	hsa05222:Small cell lung cancer	3.50E-02	7.72	2.10	2.76	5.62	2.10
	hsa04310:Wnt signaling pathway	3.54E-02	3.94	-1.20	-1.20	2.61	1.33
	hsa05160:Hepatitis C	3.89E-02	6.93	-0.22	0.20	0.95	5.98
	hsa04520:Adherens junction	3.94E-02	5.34	-0.20	-0.02	1.13	4.22
	hsa04722:Neurotrophin signaling pathway	4.61E-02	8.27	-0.36	1.07	1.83	6.43
	hsa05414:Dilated cardiomyopathy	4.81E-02	7.03	0.74	0.74	1.45	5.58
	hsa04662:B cell receptor signaling pathway	4.82E-02	3.89	0.60	0.60	1.59	2.31
	hsa05031:Amphetamine addiction	4.89E-02	5.06	-0.36	0.00	0.73	4.33
	hsa05020:Prion diseases	4.90E-02	6.57	1.06	1.06	2.93	3.64
Down-regulation	hsa05010:Alzheimer's disease	3.04E-07	23.14	1.28	0.00	1.07	22.07
	hsa05012:Parkinson's disease	4.97E-07	32.52	0.42	-0.75	1.26	31.27
	hsa05016:Huntington's disease	2.48E-06	27.58	0.58	0.00	1.02	26.56
	hsa04962:Vasopressin-regulated water reabsorption	1.73E-03	10.21	1.16	0.00	1.16	9.04
	hsa05132:Salmonella infection	1.26E-02	5.24	-0.97	-1.26	2.76	2.48

hsa04722:Neurotrophin signaling pathway	1.34E-02	6.41	-1.86	-2.51	4.48	1.93
hsa04727:GABAergic synapse	2.16E-02	10.55	-0.78	0.00	1.73	8.82

Table S5. Topological properties of the detected modules by WGCNA

	All	mod_blue	mod_brown	mod_turquoise	mod_yellow
Density	0.002	0.009	0.012	0.008	0.011
Centralization	0.009	0.022	0.052	0.025	0.026
Heterogeneity	1.161	0.743	1.265	0.861	0.773
Mean ClusterCoef	0.021	0.027	0.069	0.034	0.037
Mean Connectivity	1.152	1.080	1.083	1.233	0.787

Table S6. Functional enrichment of the WGCNA Modules.

Module	Term	Fold Enrichment	*p*-Value	Benjamini *Q*-Value
Turquoise (n = 147)	GO:0005184: neuropeptide hormone activity	33.20	1.31E-05	3.90E-03
	GO:0000502: proteasome complex	13.97	6.40E-05	1.33E-02
	GO:0005179: hormone activity	9.90	6.92E-05	1.02E-02
	GO:0051443: positive regulation of ubiquitin-protein ligase activity	11.95	1.36E-04	1.44E-01
	GO:0051351: positive regulation of ligase activity	11.46	1.66E-04	9.04E-02
	GO:0051438: regulation of ubiquitin-protein ligase activity	10.73	2.27E-04	8.27E-02
	GO:0051340: regulation of ligase activity	10.33	2.71E-04	7.44E-02
	GO:0016887: ATPase activity	4.57	2.99E-04	2.92E-02
	GO:0031398: positive regulation of protein ubiquitination	9.96	3.21E-04	7.07E-02
	GO:0031396: regulation of protein ubiquitination	8.37	7.17E-04	1.27E-01
	hsa03050: proteasome	11.51	7.87E-04	6.62E-02
Blue (n = 128)	GO:0019905: syntaxin binding	18.41	1.26E-03	2.71E-01
	GO:0000149: SNARE binding	15.42	2.11E-03	2.33E-01
	GO:0007243: protein kinase cascade	3.70	2.78E-03	9.44E-01
	GO:0016192: vesicle-mediated transport	2.90	4.13E-03	8.82E-01
	GO:0000139: Golgi membrane	5.03	6.49E-03	7.63E-01
	GO:0044451: nucleoplasm part	2.81	8.31E-03	6.02E-01
	GO:0031985: Golgi cisterna	21.26	8.44E-03	4.64E-01
	GO:0045892: negative regulation of transcription, DNA-dependent	3.42	8.45E-03	9.46E-01
	GO:0051253: negative regulation of RNA metabolic process	3.36	9.21E-03	9.09E-01
	GO:0004674: protein serine/threonine kinase activity	2.99	9.85E-03	5.63E-01
Brown (n = 89)	GO:0031090: organelle membrane	2.62	6.65E-03	6.95E-01
	GO:0012505: endomembrane system	2.67	2.57E-02	9.01E-01
	GO:0015031: protein transport	2.68	2.57E-02	1.00E+00
	GO:0045184: establishment of protein localization	2.66	2.69E-02	1.00E+00
	GO:0003924: GTPase activity	4.92	4.52E-02	1.00E+00

	GO:0005739: mitochondrion	2.16	4.77E-02	9.45E-01
Yellow (*n* = 73)	GO:0005739: mitochondrion	5.88	3.83E-14	4.48E-12
	GO:0005840: ribosome	13.72	6.24E-10	3.65E-08
	GO:0003735: structural constituent of ribosome	17.56	3.17E-09	3.49E-07
	GO:0044429: mitochondrial part	6.61	5.88E-09	2.29E-07
	GO:0016655: oxidoreductase activity acting on NADH or NADPH, quinone, or similar compound as acceptor	42.15	1.15E-08	6.35E-07
	GO:0070469: respiratory chain	26.22	1.69E-08	4.96E-07
	hsa00190: oxidative phosphorylation	16.00	2.26E-08	4.08E-07
	GO:0006412: translation	10.97	2.97E-08	7.86E-06
	GO:0033279: ribosomal subunit	17.28	3.60E-08	8.43E-07
	GO:0022900: electron transport chain	23.15	3.71E-08	4.92E-06
	GO:0030529: ribonucleoprotein complex	6.68	7.41E-08	1.44E-06
	GO:0006091: generation of precursor metabolites and energy	10.54	2.46E-07	2.18E-05
	GO:0050136: NADH dehydrogenase (quinone) activity	41.17	2.75E-07	1.01E-05
	GO:0003954: NADH dehydrogenase activity	41.17	2.75E-07	1.01E-05
	GO:0008137: NADH dehydrogenase (ubiquinone) activity	41.17	2.75E-07	1.01E-05
	GO:0016651: oxidoreductase activity acting on NADH or NADPH	24.89	2.87E-07	7.89E-06
	hsa05012: Parkinson's disease	14.45	4.71E-07	4.24E-06
	GO:0005761: mitochondrial ribosome	30.73	1.24E-06	2.08E-05
	GO:0000313: organellar ribosome	30.73	1.24E-06	2.08E-05
	hsa05010: Alzheimer's disease	11.34	2.43E-06	1.46E-05
	hsa05016: Huntington's disease	10.27	4.73E-06	2.13E-05
	GO:0005746: mitochondrial respiratory chain	23.04	5.28E-06	7.72E-05
	GO:0006119: oxidative phosphorylation	20.20	9.69E-06	6.42E-04
	GO:0044455: mitochondrial membrane part	13.77	9.74E-06	1.27E-04
	GO:0042775: mitochondrial ATP synthesis coupled electron transport	29.46	2.15E-05	1.14E-03
	GO:0042773: ATP synthesis coupled electron transport	29.46	2.15E-05	1.14E-03
	GO:0005743: mitochondrial inner membrane	7.23	2.56E-05	2.99E-04
	GO:0005198: structural molecule activity	5.12	3.19E-05	7.00E-04
	GO:0005740: mitochondrial envelope	5.87	3.59E-05	3.82E-04
	GO:0022904: respiratory electron transport chain	25.78	3.66E-05	1.62E-03
	GO:0019866: organelle inner membrane	6.72	4.29E-05	4.18E-04
	GO:0005763: mitochondrial small ribosomal subunit	54.62	4.68E-05	4.21E-04
	GO:0000314: organellar small ribosomal subunit	54.62	4.68E-05	4.21E-04
	GO:0015935: small ribosomal subunit	19.51	1.13E-04	9.40E-04

Table S7. Functional enrichment of the WGCNA Modules (MITHrIL)

Module	Term	*P*-Value	Impact Factor	Perturbation Accumulation	Raw Accumulation	Total Perturbation	Probability
turquoise (n=147)	hsa04370:VEGF signaling pathway	2.84E-03	8.92	-1.11	-0.81	1.94	6.98
	hsa04722:Neurotrophin signaling pathway	3.90E-03	6.98	-1.78	-1.80	3.90	3.09
	hsa04727:GABAergic synapse	4.82E-03	13.64	-0.78	0.00	1.57	12.07
	hsa04650:Natural killer cell mediated cytotoxicity	5.96E-03	6.91	2.27	2.80	4.08	2.82
	hsa04660:T cell receptor signaling pathway	1.44E-02	4.80	0.60	0.60	1.53	3.27
	hsa05212:Pancreatic cancer	1.57E-02	5.47	0.52	0.40	1.29	4.19
	hsa04730:Long-term depression	1.69E-02	7.94	-1.35	-1.35	3.60	4.34
	hsa04912:GnRH signaling pathway	1.85E-02	5.09	0.60	0.60	1.53	3.57
	hsa05034:Alcoholism	2.23E-02	6.18	1.64	0.83	1.95	4.23
	hsa04520:Adherens junction	3.63E-02	6.07	-0.63	-0.78	2.07	4.00
	hsa05164:Influenza A	3.79E-02	3.75	0.40	0.40	1.32	2.43
Blue (n=128)	hsa04724:Glutamatergic synapse	9.71E-03	10.42	-1.10	-1.10	2.19	8.23
	hsa04713:Circadian entrainment	9.95E-03	8.54	2.26	2.52	4.92	3.63
	hsa04810:Regulation of actin cytoskeleton	1.18E-02	5.21	-0.99	-0.30	1.24	3.97
	hsa04976:Bile secretion	1.31E-02	6.04	0.75	0.75	1.88	4.16
	hsa04725:Cholinergic synapse	1.48E-02	8.11	-1.11	0.34	2.39	5.72
	hsa04727:GABAergic synapse	1.70E-02	11.10	-0.42	-0.76	1.82	9.28
	hsa04010:MAPK signaling pathway	2.18E-02	10.79	-0.17	0.29	1.17	9.62
	hsa05131:Shigellosis	2.82E-02	8.79	0.38	0.38	1.32	7.47
	hsa04370:VEGF signaling pathway	3.21E-02	7.97	0.73	1.49	3.67	4.30
	hsa04666:Fc gamma R-mediated phagocytosis	3.30E-02	3.25	-0.60	-0.30	1.74	1.52
	hsa05152:Tuberculosis	3.44E-02	7.39	1.53	2.15	4.87	2.51
	hsa04390:Hippo signaling pathway	4.01E-02	5.63	-1.18	-0.84	2.86	2.77
	hsa05120:Epithelial cell signaling in Helicobacter pylori infection	4.06E-02	6.23	0.57	0.57	1.95	4.27
	hsa05145:Toxoplasmosis	4.53E-02	8.35	1.26	1.26	5.10	3.25
	hsa05221:Acute myeloid leukemia	4.75E-02	6.19	0.31	0.49	1.58	4.61
brown (n=89)	hsa04610:Complement and coagulation cascades	9.32E-03	6.50	-1.20	-1.20	4.18	2.32
	hsa05133:Pertussis	1.17E-02	5.50	-0.87	-0.87	3.26	2.24

	hsa04310:Wnt signaling pathway	2.40E-02	6.14	-1.20	-1.20	4.50	1.65
	hsa04151:PI3K-Akt signaling pathway	4.50E-02	7.58	-1.28	0.60	3.37	4.21
	hsa04724:Glutamatergic synapse	4.68E-02	6.13	-0.69	-0.69	1.81	4.32
yellow (n=73)	hsa05010:Alzheimer's disease	1.82E-06	24.27	0.86	0.00	1.10	23.17

Table S8. Connectivity strength of intra-hub genes in the functional enriched modules. For module turquoise and blue (>100 genes), the top 10 connected genes (k_{within}) were listed. For module brown and yellow (<100 genes), the top 5 connected genes (k_{within}) were listed.

Module	Gene	k_{Total}	k_{Within}	k_{Out}	k_{Diff}
turquoise (n=147)	*SNURF*	5.99	4.85	1.14	3.71
	CDC42	6.39	4.52	1.87	2.66
	TAC1	6.89	4.45	2.44	2.02
	NSF	5.37	4.26	1.11	3.15
	LOC643668	6.14	4.25	1.89	2.37
	TM6SF1	4.11	3.32	0.79	2.53
	ACP1	4.42	3.31	1.11	2.20
	RTN4	4.22	3.30	0.92	2.38
	CRYM	4.13	3.18	0.96	2.22
	C6ORF66	5.42	3.10	2.32	0.78
blue (n=128)	*PKD1*	4.56	3.81	0.75	3.06
	KIAA0664	4.65	3.73	0.92	2.81
	OGDH	5.30	3.55	1.75	1.80
	SIN3B	5.72	2.98	2.74	0.24
	B4GALNT4	3.57	2.87	0.69	2.18
	TAOK2	3.76	2.71	1.05	1.67
	BAT2	4.36	2.60	1.76	0.84
	SNPH	2.64	2.45	0.18	2.27
	CACNA1A	3.79	2.42	1.36	1.06
	ATXN7L3	2.64	2.38	0.26	2.12
brown (n=89)	*ZNF394*	6.06	5.54	0.53	5.01
	SHROOM4	6.07	5.42	0.65	4.77
	GRIPAP1	5.73	5.27	0.46	4.81
	XRCC2	5.73	5.22	0.51	4.72
	HYPK	5.47	4.83	0.65	4.18
yellow (n=73)	*HINT1*	4.73	2.64	2.09	0.55
	TXN	3.60	2.50	1.10	1.40
	MRPS18C	2.91	2.48	0.43	2.04
	TMEM126A	3.02	2.09	0.94	1.15
	NDUFA12	2.21	2.06	0.16	1.90
